# The Roles of Molecular Chaperones Interacting with the σ^70^ Factor in Global Transcription of the *Escherichia coli* Genome

**DOI:** 10.3390/genes17060621

**Published:** 2026-05-29

**Authors:** Jianlu Jiao, Dan Wu, Xiaoli Lv, Morigen Morigen

**Affiliations:** 1 Inner Mongolia Key Laboratory for Molecular Regulation of the Cell, School of Life Sciences, Inner Mongolia University, Hohhot 010020, China; jianlujiao2026@126.com (J.J.); wudan_1024@sina.com (D.W.); 2 Academy of Science and Technology, Chuxiong Normal University, Chuxiong 675000, China; 3 Department of Pharmacology of Pharmaceutical College, Inner Mongolia Medical University, Hohhot 010110, China; lvxiaoli@immu.edu.cn

**Keywords:** molecular chaperones, σ^70^ factor, interactions, transcriptomes, *E. coli*

## Abstract

**Background/Objectives:** The σ factor of bacterial RNA polymerase (RNAP) directs promoter recognition, recruits RNAP to initiate transcription, and is released from the elongation complex to participate in subsequent rounds of initiation. However, the dynamic recycling mechanism of the primary σ factor, σ^70^ (RpoD), during transcription in *Escherichia coli* remains poorly understood. **Methods**: We employed in vivo and in vitro interaction assays to screen for σ^70^-interacting partners under different growth conditions. Protein localization studies were performed using fluorescence microscopy. The transcriptomic profile of Δ*clpB*, Δ*dnaK,* Δ*htpG*, or Δ*yhgF* mutant was assessed by RNA-seq. **Results**: The molecular chaperones ClpB, DnaK, HtpG, and the RNA-binding protein YhgF interacts with RpoD both in vivo and in vitro, and the interaction in vivo is growth medium-dependent (LB vs. ABTGcasa). During exponential growth, each of these proteins co-localizes with the nucleoid. The transcriptome profile in Δ*clpB*, Δ*htpG* or Δ*yhgF* mutant is mutant-specific to some extent; differentially expressed genes (DEGs) associated with amino acid metabolism and lipopolysaccharide biosynthesis are down-regulated in Δ*clpB*, Δ*htpG* or Δ*yhgF* mutant in a manner that is growth medium-dependent, in agreement with the medium-dependent interaction of RpoD with the chaperones and YhgF. In contrast, the absence of DnaK resulted in delays to initiation of replication with a slow growth, and decreases cell motility, accompanied by down-regulated flagellar assembly and up-regulated amino acid metabolism genes. In summary, ClpB, DnaK, HtpG, and YhgF may regulate transcription by directly interacting with σ^70^. The σ factor recycling guides global transcription to select genes for transcription and subsequently allows cells to cope with the changing environments by responding to the nutrient level as a signal.

## 1. Introduction

In all organisms, transcription is carried out by DNA-dependent RNA polymerases (RNAP) [1]. This fundamental process, through which genetic information stored in DNA is converted into various RNA molecules, including rRNA, tRNA, mRNA, and non-coding RNA, is among the most tightly regulated cellular activities [2]. In bacteria, a single RNAP, composed of α^2^, β, β’, and ω subunits, catalyzes transcription elongation [3,4,5]. The RNAP core enzyme is converted into an RNAP holoenzyme by the association of a σ factor. The σ factors serve as dissociable subunits, facilitating transcription initiation by recognizing and binding specific DNA sequences in promoters [6,7,8]. *Escherichia coli* possesses seven σ subunits, and each recognizes a specific set of promoters to activate the transcription of corresponding genes [9]. During the exponential growth, the primary housekeeping σ factor responsible for transcribing the majority of *E. coli* genes is the σ^70^ factor [10,11], encoded by the *rpoD* gene [12]. The σ^70^ factor contains several conserved structural domains: σR1.1, σR2.1, σR2.4, σR3, σR3.2, and σR4.2 [13]. The σR2.1, σR2.4, and σR4.2 domains mediate the coordinated interaction of RNAP with target promoters [14]. Specifically, σR2.4 recognizes and binds to the -10-promoter element, while σR4.2 binds to the -35 element. Following promoter recognition, an approximately 14 bp region of DNA is unwound at the AT-rich −10 box, and transcription is initiated at the +1 start site [8]. The σR1.1 domain functions as the “gatekeeper” of the RNAP active center, permitting access to promoter DNA but preventing non-specific binding to non-promoter sites [15]. The σR3.2 domain contacts the template DNA strand during initiation [16,17,18]. When the nascent RNA transcript reaches a length of more than 15 nucleotides, σR3.2 can be replaced by the elongating RNA strand. This disrupts its interaction with the template and triggers the dissociation of σ^70^ from the transcribing RNAP, a critical step for promoter escape [19].

Molecular chaperones are pervasive and incredibly adaptable dynamic proteins that interact with and protect substrate proteins by preventing protein misfolding under unfavorable conditions. They are classified into the main functional categories of holdases, foldases, and disaggregases [20,21]. Holdases, such as trigger factor (TF) and IbpA/IbpB, bind to folding intermediates to prevent aggregation [22,23]. Foldases, including DnaK, GroEL, and HtpG, facilitate the ATP-dependent refolding of misfolded proteins and assist in the folding of nascent polypeptide chains [24]. Disaggregases, exemplified by ClpB, solubilize protein aggregates [25]. Emerging evidence indicates that several chaperones can modulate transcription by influencing the interaction between σ factors and the RNAP core enzyme, thereby altering promoter selectivity and transcriptional activity [26]. For instance, in *Actinomycetes* sp., the chaperone RbpA targets the σR2 domain of σ factors, regulates the conformational state of σB and promotes the RNAP holoenzyme [27,28]. In *Caulobacter crescentus*, the GcrA chaperone associates with the σ^70^-RNAP holoenzyme at promoters and stimulates the transcription of specific methylated genes through interaction with the σR2 region of σ^70^ [29]. In *E. coli*, the bacteriophage T4-encoded protein AisA binds to σ^70^ and remodels the conformation of σR3.2 and σR4 domains, thereby inhibiting their normal contact with promoter DNA [30]. Additionally, the chaperone-like protein RapA has been shown to facilitate the release of RNAP from post-transcription complexes, promoting transcription reinitiation [31]. In the classic σ cycle model, the σ factor dissociates from RNAP after transcription initiation, and rebinds for subsequent rounds of transcription. While this model holds true in most contexts, σ factors may occasionally remain associated with elongating RNAP complexes and play regulatory roles under certain conditions [1].

Despite these advances, the precise roles of molecular chaperones that interact with σ factors in transcription regulation remain poorly understood. Several key questions about the σ^70^ cycle in *E. coli* have yet to be addressed: How does σ^70^ dissociate from the transcribing RNAP after initiation? How is it recruited for subsequent rounds of transcription? And what signals govern this cycle? To address the questions, we performed the following investigations: (i) to identify σ^70^-interacting proteins in *E. coli* under different growth conditions; (ii) to characterize the global transcription patterns in *clpB*, *dnaK*, *htpG*, or *yhgF* mutant; (iii) to find the correlation between the global transcription pattern with phenotypic outcomes; and (iv) to propose a model for how these proteins function in σ^70^ recycling and subsequently in growth medium-specific global transcription. In this study, we demonstrate that the molecular chaperones ClpB, DnaK, HtpG, and the RNA-binding protein YhgF interact with σ^70^ in a growth medium-dependent manner, thereby influencing global transcription. These findings suggest that these proteins may play a role in the σ^70^ recycling cycle, with nutrient levels serving as a key signaling cue that modulates this process and enables adaptive transcriptional responses to environmental changes.

Furthermore, the σ^70^ recycling might be an important clue to find the clinical implication since pathogenic *E. coli* requires rapid transcriptional reprogramming to cope with the host environments [32]. Dysregulation of the σ factor activity is found to be linked to bacterial virulence, biofilm formation, and antibiotic tolerance [33]. For instance, mutations affecting σ^70^ function can alter the expression of genes involved in lipopolysaccharide (LPS) biosynthesis, flagellar assembly, and toxin production, all of which are critical for pathogenicity [34,35]. It is also shown that molecular chaperones such as DnaK and ClpB support bacterial survival under host-associated stresses, including fever, oxidative bursts, and nutrient limitation [36,37]. Targeting chaperone networks can sensitize multidrug-resistant *E. coli* to antibiotics, suggesting that σ^70^–chaperone interactions may represent a novel antimicrobial target [38].

## 2. Materials and Methods

### 2.1. Bacterial Strains, Growth Conditions, and Plasmids

All strains employed in this study, including BW25113 (wild type, WT), are derived from *Escherichia coli* K-12 and listed in Table 1, unless otherwise specified. Cells were grown at 37 °C in either Luria–Bertani (LB) or ABTGcasa medium [39]. ABTGcasa is AB minimal medium [40] (6 g/L Na_2_HPO_4_, 2 g/L (NH_4_)_2_SO_4_, 3 g/L KH_2_PO_4_, 3 g/L NaCl) supplemented with 10 μg/mL thiamine, 0.2% glucose, and 0.5% Casamino Acids. Plasmids used are listed in Supplementary Materials Table S1, and primers in Supplementary Materials Table S2. The *rpoD*, *clpB*, *yhgF*, *htpG*, or *dnaK* gene was amplified with a pair of primers targeting the gene of interest (primer 13–22 listed in Supplementary Materials Table S2), using the genomic DNA of strain BW25113 as a template. The PCR fragment of each gene was inserted at the *Hind*III and *BamH*I sites of plasmid pET28a/pCA24N/pUT18/pKNT25, accordingly, and the desired plasmids were obtained as listed in Supplementary Materials Table S1. The *neo* (*kan^R^*) gene was PCR amplified using the pKD4 plasmid as a template and a pair of primers 29 and 30 (otherwise listed in Supplementary Materials Table S2), then inserted into BW25113 to replace the chromosomal *dnaK*, *clpB*, *htpG*, or *yhgF* gene through homologous recombination by one-step chromosomal gene inactivation method [41], resulting in BW25113Δ*dnaK*:: *neo* (*kan^R^*), BW25113Δ*clpB*::*neo* (*kan^R^*), BW25113Δ*htpG*:: *neo* (*kan^R^*) or BW25113Δ*yhgF*::*neo* (*kan^R^*) mutant (Supplementary Materials Figure S1). All plasmids were constructed using the DH5α strain of *E. coli* as the recipient cells. BL21(DE3) was used for the expression and purification of the His_6_-fused proteins. All PCR fragments were sent to Sangong Biotech Company (Shanghai, China) for sequencing.

### 2.2. GFP-Trap Immunoprecipitation and Mass Spectrometry Analysis

Immunoprecipitation for cells carrying pCA24N-RpoD-GFP was performed by using GFP-Trap^®^ A kit (ChromoTek, Planegg, Germany), following the manufacturers’ instructions. Cells were washed with PBS, and lysed in the NP40 lysis buffer (10 mM Tris/HCl (pH = 7.5), 150 mM NaCl, 0.5 mM EDTA, and 0.5% NP-40) with CPI (1× cOmplete Protease inhibitor cocktail) (Sigma Aldrich, St. Louis, MO, USA). The cell lysate (supernatant) was collected by centrifugation at 16,000× *g*, 4 °C. GFP-Trap magnetic or agarose beads were equilibrated in dilution/wash buffer (10 mM Tris/HCl (pH = 7.5), 150 mM NaCl, 0.5 mM EDTA, CPI). The cell lysate was added to the equilibrated GFP-trap^®^ beads and incubated with gentle end-to-end rotations for 12 h at 4 °C. The beads containing proteins were collected and washed three times in 500 μL of ice-cold dilution buffer (without CPI) [44]. The proteins bound on beads were eluted with 2 × SDS sample buffer containing 2% SDS, 20% glycerol, 2% β-mercaptoethanol, 0.5 M Tris/HCl, pH 6.8 and 0.1 mg/mL bromophenol blue dye. The eluted proteins were separated by SDS-PAGE, stained in Coomassie blue, and followed by destaining. The protein bands were cut and sent for mass spectrometry analysis and Western blotting probe with GFP antibody (HT801, TransGen Biotech, Beijing, China) was performed as described previously [45] (Supplementary Materials Figure S2A). The experiment included the pCA24N plasmid as a negative control and pCA24N-*uvrY* as a positive control.

### 2.3. Surface Plasmon Resonance (SPR) Assay

Strain BL21(DE3) carrying pET28a-*rpoD*, pET28a-*clpB*, pET28a-*yhgF*, pET28a-*htpG* or pET28a-*dnaK* (Supplementary Materials Table S1) was exponentially grown in LB to express the recombinant protein with His-tag on the N terminus. The recombinant RpoD, ClpB, YhgF, HtpG or DnaK protein with His-tag was purified by nickel affinity chromatography (Ni-NTA) which specifically binds His-tag of the recombinant protein. The running buffer used during the experiments was 1× HBS-EP + buffer (10× 0.1 M HEPES; 1.5 M NaCl; 0.03 M EDTA; 0.5% P20). All reagents were filtered with 0.22 filter membrane before use. The purified RpoD protein was subsequently diluted using sodium acetate at pH levels of 4.0, 4.5, and 5.0. Subsequently, RpoD was immobilized on a CM5 sensor chip (GE Healthcare, Uppsala, Sweden) using an amine coupling kit (GE Healthcare, Uppsala, Sweden) according to the manufacturer’s instructions for the Biacore-T200 (GE Healthcare, Uppsala, Sweden). Protein ClpB, YhgF, HtpG or DnaK analytical solutions with different concentrations were prepared according to the gradient, which was then flowed through the chip coupled with RpoD in sequence from low to high. All binding experiments were performed at 25 °C. As a control, each sample was passed over a reference flow cell containing no RpoD. The sensor chip could be regenerated with three washes of 3 μL of 20 mM glycine (pH = 2.0). The change in refractive index caused by the binding of molecules to the SPR sensor disk surface led to a shift in the SPR angle. By monitoring the changed SPR angle, the kinetic binding and dissociation constant, affinity, and specificity of the analyte could be obtained. All the data obtained were analyzed by Biacore T200 Evaluation Software v3.2.1 (GE Healthcare, Uppsala, Sweden) and the binding levels (increase in relative response from the baseline) were obtained. The experiment was essentially done as described by Li et al. [46].

### 2.4. Bacterial Two-Hybrid Assay

The bacterial two-hybrid (BACTH) system was employed to detect protein–protein interactions, using the T18 and T25 fusion plasmids pUT18 and pKNT25 (BACTH System Kit (EUK001), Euromedex, Souffelweyersheim, France). The *rpoD*, *clpB*, *htpG*, *dnaK*, or *yhgF* genes were PCR-amplified and cloned into the pKNT25 and pUT18 plasmids. Various plasmid combinations, including pKNT25-*rpoD*/pUT18-*rpoD*, pKNT25-*yhgF*/pUT18-*yhgF*, pKNT25-*rpoD*/pUT18-*rpoD*, pKNT25-*clpB*/pUT18-*clpB*, pKNT25-*rpoD*/pUT18-*rpoD*, pKNT25-*htpG*/pUT18-*htpG*, pKNT25-*rpoD*/pUT18-*rpoD*, and pKNT25-*dnaK*/pUT18-*dnaK* (Supplementary Materials Table S1), were co-transformed into strain BTH101. All plasmids used are listed in Supplementary Materials Table S1. Transformants carrying each plasmid pair were plated on LB or ABTGcasa (Supplementary Materials Table S6) agar (1.5%) plates supplemented with ampicillin (100 μg/mL), kanamycin (50 μg/mL), 5-bromo-4-chloro-3-indolyl β-D-galactopyran oside (X-gal, 40 μg/mL), and isopropyl-beta-D-thiogalactopyranosid (IPTG, 0.5 mM), and incubated at 30 °C for 42–72 h [45]. The BACTH system has an adenylyl cyclase activation region. If there is an interaction between the two proteins, adenylyl cyclase is activated, which in turn activates the cAMP signaling molecule. cAMP catalyzes the expression of β-galactosidase (*lacZ*) and appears blue on X-gal and IPTG plates [43]. When two proteins produced from the plasmid pair interact in vivo, blue colonies form on the plates; otherwise, the transformant colonies are white. Empty vectors (pUT18 and pKNT25) were used as negative controls, while pKNT25-*mreB* and pUT18-*torR* were used as positive controls in the bacterial two-hybrid assay.

### 2.5. Co-Localization of RpoD, Chaperones, and YhgF with Nucleoid

Plasmids pCA24N-*rpoD*-gfp, pCA24N-*clpB*-gfp, pCA24N-*yhgF*-gfp, pCA24N-*htpG*-gfp, or pCA24N-*dnaK*-gfp (Supplementary Materials Table S1) were introduced into wild-type BW25113, Δ*clpB*, Δ*yhgF*, Δ*htpG*, or Δ*dnaK* cells, respectively. Then the cells were exponentially grown at 37 °C in ABTGcasa medium [47] supplemented with required antibiotics. The cells were harvested by centrifugation at 16,000× *g*, 4 °C for 10 min (min), and fixed with 1 mL of 70% ethanol and stored at 4 °C for further use after a wash with pre-chilled 1 × TE (1 M Tris-HCl and 0.5 M EDTA, pH = 8.0). The fixed cells were stained in Hoechst 33258 (final concentration of 3 μg/mL (Invitrogen, Carlsbad, CA, USA), 0.02 M Tris-HCl buffer supplemented with 130 mM NaCl) for 30 min or longer. A confocal fluorescence microscope (ZEISS LSM710, Carl Zeiss AG, Jena, Germany) was used to observe, visualize and photograph green GFP recombinant protein and blue nucleoid stained in Hoechst 33258 as described previously [45].

### 2.6. Total RNA Extraction and RT-qPCR

WT and mutants strains (Δ*clpB*, Δ*yhgF*, Δ*htpG*, or Δ*dnaK*) were cultured at 37 °C to exponential phase (an optical density at 600, OD_600_ = 0.3), then harvested by centrifugation at 4 °C. Total RNAs of cells were extracted with Trizol reagent (TRIzol^TM^ Plus, Invitrogen, Carlsbad, CA, USA), according to the instructions from the manufacturer. RNA integrity was assessed by electrophoresis in 1% agarose gel stained with 1 μL/mL 4S Red Plus Nucleic Acid Stain (Sangon Biotech, Shanghai, China). The 23S/16S ratios of all samples measured using an Agilent Bioanalyzer (Agilent Technologies, Santa Clara, CA, USA) were found to be about 2:1. RNA purity was determined using the NanoDrop 2000C spectrophotometer (Thermo Fisher Scientific^TM^, Waltham, MA, USA) by finding the A260/A230 and A260/A280 ratios. The A260/A280 ratios of all samples were 1.9–2.1 and the A260/A230 ratios were 2.0–2.1. All samples meet the requirements of RNA integrity and purity for the reverse transcriptional quantitative PCR (RT-qPCR) analysis. RT-qPCR was performed as previously described [48], using the primers listed in Supplementary Materials Table S3 (synthesized by Sangon Biotech). The mRNA levels of target genes were calculated relative to 16S rDNA for each strain; additionally, the mRNA level of each gene in each mutant relative to that in WT cells was calculated.

### 2.7. Bioinformatics Analysis of Sequencing Data

RNA-seq was carried out on wild-type and mutant strains (Δ*clpB*, Δ*yhgF*, Δ*htpG*, and Δ*dnaK*) for comparative transcriptomic analysis. Ten sequencing libraries were generated on an Illumina platform (Illumina, San Diego, CA, USA). The raw reads were 39.24 million, and 39.01 million clean reads were obtained after filtration. The percentages of Q20 bases and Q30 bases were more than 93.87% and 99.24%, respectively. The GC content among the total bases was 51.46% to 44% (Supplementary Materials Table S6). The percentages of clean reads from all samples mapped to the reference genes (GCF_000750555.1) using Bowtie2 software (version 2.5.4) [49] were between 92.05% and 95.89%. The square of the Pearson correlation coefficient (R^2^) for each sample was 0.854–0.945. These results suggested that the RNA-Seq data were credible and could be used for further analysis. Differential expression between mutants and WT was detected using DEseq2 (version 4.0.4) [50]. Genes with fold change > 1 and adjusted *p*-value < 0.05 were identified as differentially expressed genes (DEGs). Gene Ontology (GO) functional analysis provides GO functional classifications and annotations for DEGs. Various genes usually cooperate with each other to exercise their biological functions. A pathway-related database was therefore obtained based on Kyoto Encyclopedia of Genes and Genome (KEGG) results (http://www.genome.jp/kegg/, accessed on 1 May 2026).

### 2.8. Flow Cytometry

Exponentially growing cells of WT and mutant strains (Δ*clpB*, Δ*yhgF*, Δ*htpG*, or Δ*dnaK*) were cultured in LB (OD_600_ = 0.15) or ABTGcasa medium (an optical density at 450 nm, OD_450_ = 0.15), then treated with rifampicin (300 μg/mL) and cephalexin (10 μg/mL) for 3–5 generations. Rifampicin prevents initiation of DNA replication but allows ongoing replication finish and cephalexin inhibits cell division [51,52]. Then cells were analyzed by flow cytometer (BD LSRFortessa^TM^, BD Biosciences, San Jose, CA, USA) after being washed and fixed in 1 × TE and 70% ethanol, stained in Hoechst 33258 (Invitrogen) for 30 min and washed in Tris-HCl NaCl buffer (pH = 7.5) [52]. Preparation of standard samples and calculation of the average number of origins per cell (A.O.) were performed as described [47,53,54,55].

### 2.9. Growth Conditions and Determination of Doubling Time

Cells from WT and mutant strains (Δ*clpB*, Δ*yhgF*, Δ*htpG*, or Δ*dnaK*) were cultured at 37 °C in LB or ABTGcasa medium [39]. Growth was monitored by measuring optical density at 600 nm (LB) or 450 nm (ABTGcasa), using an ultraviolet spectrophotometer (UV-1800, Shimadzu, Kyoto, Japan). Readings were taken at various time points, beginning at OD_600_/OD_450_ = 0.05 and continuing until OD_600_/OD_450_ > 0.5. Doubling time for each mutant was calculated as previously described [56].

### 2.10. Cell Motility Assay

Cell motility assays were performed as mentioned previously [57]. Overnight cultures of WT and mutant strains (Δ*clpB*, Δ*yhgF*, Δ*htpG*, or Δ*dnaK*) were grown at 37 °C in LB or ABTGcasa medium with required antibiotics. A total of 1 μL for each culture was then stabbed into LB or ABTGcasa semi-solid plate (0.3% agar) supplemented with 0.2% arabinose and required antibiotics. After 16 h of incubation, cell halos were measured and photographed [57]. Cell halo diameter was measured using ImageJ (version 1.x; https://imagej.net/software/imagej/; accessed on 1 May 2026).

## 3. Results

### 3.1. RpoD (σ^70^ Factor) Interacts with the ClpB, HtpG, DnaK Molecular Chaperones and YhgF Both In Vitro and In Vivo

**Figure 1:**
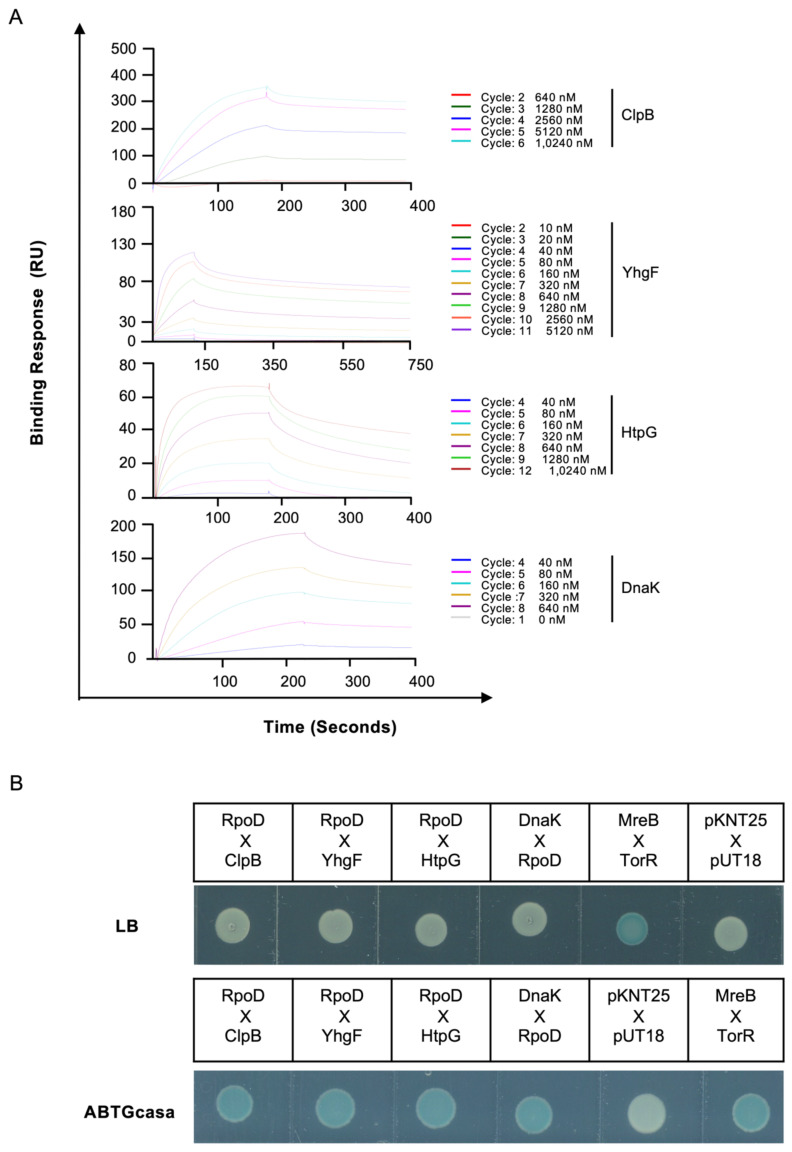
RpoD (σ^70^ factor) interacts with ClpB, YhgF, HtpG, and DnaK both in vitro and in vivo. (**A**) Interactions of RpoD with ClpB, YhgF, HtpG, and DnaK were analyzed as described in Materials and Methods by SPR spectroscope (Biacore-T200, GE Healthcare, Uppsala, Sweden) with different RpoD concentrations as indicated. The binding parameters analyzed by SPR are summarized in Table 3. All experiments were performed in duplicate. (**B**) BACTH analysis was performed on LB and ABTGcasa medium, respectively. The detailed plasmid constructions, transformations and observation were carried out as mentioned in Materials and Methods. The protein couple tested and growth media used are as indicated, and the representatives of results from three repeated experiments are shown.

Through immunoprecipitation using the RpoD-GFP-Trap, the molecular chaperone ClpB, HtpG, DnaK or an RNA-binding protein YhgF [58] was pulled down (Supplementary Materials Figure S2A, Supplementary Materials Table S4), suggesting that RpoD may interact with ClpB, HtpG, DnaK, or YhgF (Table 2). To validate the interactions, a Surface Plasmon Resonance (SPR) analysis was conducted. The *E. coli* expression strain BL21(DE3) was transformed with pET28a-*rpoD*, pET28a-*clpB*, pET28a-*yhgF*, pET28a-*htpG* or pET28a-*dnaK* [59]. His-tagged recombinant proteins were subsequently purified from each transformant by nickel affinity chromatography (Ni-NTA) (Supplementary Materials Figure S2B). Subsequently, the interaction of the purified ClpB, YhgF, HtpG or DnaK protein with RpoD were detected through the SPR analysis. In the SPR analysis, RpoD was immobilized onto the CM5 chip as the ligand, and the analyte protein (ClpB, YhgF, HtpG or DnaK) with the concentration gradients as shown in Figure 1A was flowed over the chip in descending order, respectively. When the analyte is passed over an immobilized ligand through a microfluidic channel, an interaction between the ligand and analyte leads to the formation of a complex structure. This causes a change in the mass on the sensor chip surface and, thereby, a change in the SPR signal of the sensorgram (Response Unit, RU) [60]. As shown in Figure 1A, the interactions between ClpB, YhgF, HtpG, or DnaK and RpoD were reflected by a change in the SPR signal during the association phase. This change/difference in the SPR signal is used to derive kinetic constants for both complex formation (association) and its dissociation in a particular molecular interaction between a ligand and an analyte (Table 3). The equilibrium dissociation constant (K_D_) was calculated as the ratio of the dissociation rate constant (kd) to the association rate constant (ka). A smaller value of K_D_ represents a stronger protein–protein interaction. Notably, the interacting intensity of RpoD with ClpB, YhgF, HtpG, or DnaK was different (Table 3). The affinity of analytes for RpoD followed the order of YhgF (3.809 × 10^−8^) > DnaK (1.056 × 10^−8^) > HtpG (1.882 × 10^−7^) > ClpB (1.671 × 10^−7^). These results indicate that RpoD interacts with ClpB, HtpG, YhgF or DnaK in vitro.

To further confirm the interaction of RpoD with ClpB, HtpG, YhgF, or DnaK in vivo, we performed the bacterial two-hybrid (BACTH) assay. Empty vectors (pUT18 and pKNT25) were used as negative controls, while pKNT25-*mreB* and pUT18-*torR* [56] were used as positive controls in the bacterial two-hybrid assay. The use of two distinct growth media is intended to demonstrate the effects of growth medium composition and growth rate on transcription and protein–protein interactions. It should be emphasized that the transformants would be blue if an interaction between two proteins tested is present; otherwise, they would be white. On LB plates, each combination of RpoD with YhgF, ClpB, HtpG, or DnaK yielded white colonies, whereas the same combinations produced blue colonies on ABTGcasa plates (Figure 1B). The negative control remained white, and the positive control was blue under both conditions (Figure 1B). These results indicate that the σ^70^ factor (RpoD) interacts with ClpB, YhgF, HtpG or DnaK in vivo in a manner that is growth medium-dependent.

### 3.2. RpoD, ClpB, HtpG, DnaK, or YhgF Co-Localizes with the Nucleoid During Exponential Growth

**Figure 2:**
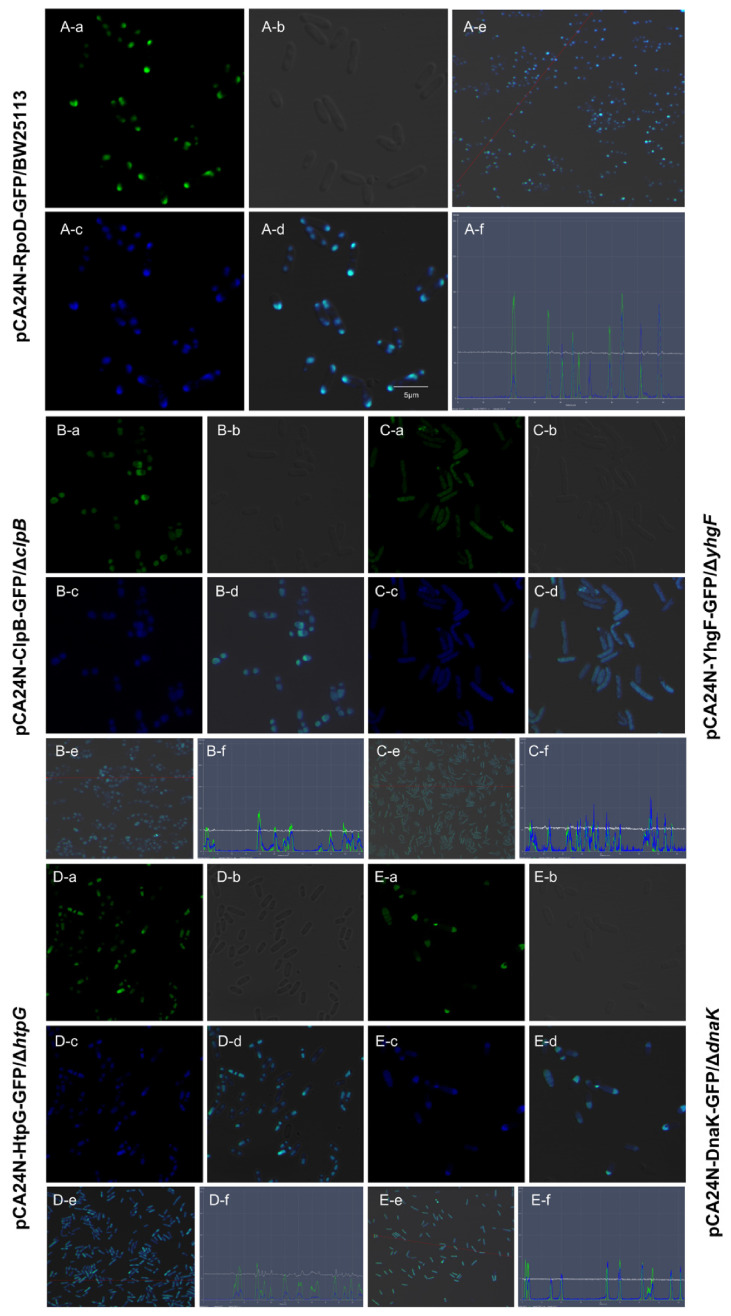
RpoD, ClpB, YhgF, HtpG and DnaK co-localize with nucleoids. (**A**) pCA24N-*rpoD*-gfp/BW25113, (**B**) pCA24N-*clpB*-gfp/Δ*clpB*, (**C**) pCA24N-*yhgF*-gfp/Δ*yhgF*, (**D**) pCA24N-*htpG*-gfp/Δ*htpG*, or (**E**) pCA24N-*dnaK*-gfp/Δ*dnaK* cells were exponentially grown at 37 °C in ABTGcasa medium with induction of IPTG (0.1 mM). The cells were harvested and fixed in 70% ethanol after a wash, and nucleoids were stained with Hoechst33258 (blue). The green fluorescence from GFP (**a**), brightfield cells images (**b**), blue fluorescence from Hoechst33258 (**c**), and overlap of green and blue fluorescence (**d**) are shown. A lower-magnification wide-field image (**e**) displays multiple cells, with a red line indicating the position used for line-scan analysis. The corresponding fluorescence intensity histogram (**f**) plots the pixel intensity profiles of GFP (green line) and Hoechst 33258 (blue line) along the red line in (**e**), demonstrating the spatial co-localization of the GFP-tagged protein with the nucleoid. All images were acquired using confocal fluorescence microscope (ZEISS, LSM710, Oberkochen, Germany) (100× objective) and ZEISS software (version 3.12).

Transcription initiation, elongation and termination are significantly influenced by bacterial chromatin proteins which organize the DNA structure [61], and the transcriptional machinery, in turn, may contribute to nucleoid organization [62]. In eukaryotic cells, chaperones have been shown to mediate nucleosome assembly [63]. To investigate whether the RpoD-interacting proteins ClpB, HtpG, DnaK, and YhgF play a role in transcription control, we examined their subcellular localization in exponentially growing cells. Plasmid pCA24N-*rpoD*-gfp (Supplementary Materials Table S1) was introduced into BW25113 (wild-type, WT) strain. In cells grown exponentially in ABTGcasa medium at 37 °C, confocal microscopy revealed that the green fluorescence signal of RpoD-GFP (Figure 2(A-a)) overlapped with the blue fluorescence of Hoechst-stained DNA (Figure 2(A-c,A-d)), indicating co-localization of RpoD with the nucleoid. This finding is consistent with previous reports [64,65]. No specific localization was observed in cells expressing the empty GFP vector (Supplementary Materials Figure S3). Confocal images were analyzed by drawing an intensity line profile of the cells in the transverse section, and fluorescence intensities of each channel were plotted as histograms using Zeiss software [66] (Figure 2(A-f)). The two independent emission wavelengths of GFP and Hoechst overlapped in space, showing that RpoD-GFP and the nucleoid co-localize within the same region. The finding indicates that RpoD, the σ factor of RNAP, and the nucleoid are co-localized. In contrast, when the same assay was performed with cells grown exponentially in LB medium at 37 °C, no co-localization between RpoD-GFP and the nucleoid was detected (Supplementary Materials Figure S4A).

Similarly, plasmids pCA24N-*clpB*-gfp, pCA24N-*yhgF*-gfp, pCA24N-*htpG*-gfp or pCA24N-*dnaK*-gfp (Supplementary Materials Table S1) were introduced into Δ*clpB*, Δ*yhgF*, Δ*htpG* or Δ*dnaK* cells. In cells grown exponentially in ABTGcasa at 37 °C, we detected an overlap between the green fluorescence signal of ClpB, YhgF, HtpG or DnaK and the blue fluorescence signal of Hoechst-stained nucleoids (Figure 2(B-d,C-d,D-d,E-d)). The histogram of fluorescence intensities of each channel showed that the two independent emission wavelengths of GFP and Hoechst overlapped in space (Figure 2(B-f,C-f,D-f,E-f)). These results indicate that ClpB, YhgF, HtpG or DnaK co-localizes with the nucleoid in exponentially growing cells. In contrast, when the same co-localization assay was performed using cells grown exponentially in LB medium at 37 °C, no overlap between the GFP-tagged proteins and Hoechst-stained nucleoids was observed (Supplementary Materials Figure S4). These findings demonstrate that the nucleoid association of ClpB, YhgF, HtpG, and DnaK is also growth medium-dependent, further supporting that nutrient conditions modulate the subcellular localization of these proteins.

To determine the effects of ClpB, YhgF, HtpG, and DnaK on the co-localization of RpoD with nucleoid, plasmid pCA24N-*rpoD*-gfp was transferred into Δ*clpB*, Δ*yhgF*, Δ*htpG*, or Δ*dnaK* cells, and co-localization with the nucleoid was detected as described above. In Δ*clpB*, Δ*yhgF*, or Δ*htpG* cells (Supplementary Materials Figure S5(A-d,A-f,B-d,B-f,C-d,C-f)), co-localization of RpoD with the nucleoid was similar to that observed in WT cells (Figure S5(A-e,A-f)), indicating that ClpB, YhgF, and HtpG do not affect the co-localization of RpoD with the nucleoid. However, the RpoD-GFP foci were formed in Δ*dnaK* mutant (Supplementary Materials Figure S5(D-a)), and the green signal (RpoD-GFP) (Supplementary Materials Figure S5(C-f)) was much weaker (barely visible in some cases) than that in WT cells (Figure 2(A-f)), indicating that the co-localization of RpoD with the nucleoid is weakened in Δ*dnaK* mutant. These results suggest that DnaK likely plays a role in the co-localization of σ factor with the nucleoid, as its absence led to impaired co-localization of RpoD with the nucleoid.

### 3.3. Global Transcriptional Change in ΔclpB, ΔyhgF, ΔhtpG, or ΔdnaK Cells Is Growth Medium-Dependent

As presented above, RpoD interacts with ClpB, YhgF, HtpG, or DnaK both in vivo and in vitro in a growth medium-dependent manner (Figure 1). To investigate the roles of chaperones and YhgF in transcription, we performed transcriptome profiling of Δ*clpB*, Δ*yhgF*, Δ*htpG*, or Δ*dnaK* cells relative to that of the WT by the RNA-seq technique. Cells were harvested during exponential growth in both ABTGcasa and LB medium, as previously described [67]. The differently expressed genes (DEGs) were identified according to the criteria outlined in the Materials and Methods. To validate the RNA-seq data, the relative RNA levels of several selected up- or down-regulated DEGs were measured via reverse transcriptional quantitative PCR (RT-qPCR). The transcriptional patterns of the genes tested were largely consistent with the RNA-seq results (Supplementary Materials Figures S8 and S9).

**Figure 3:**
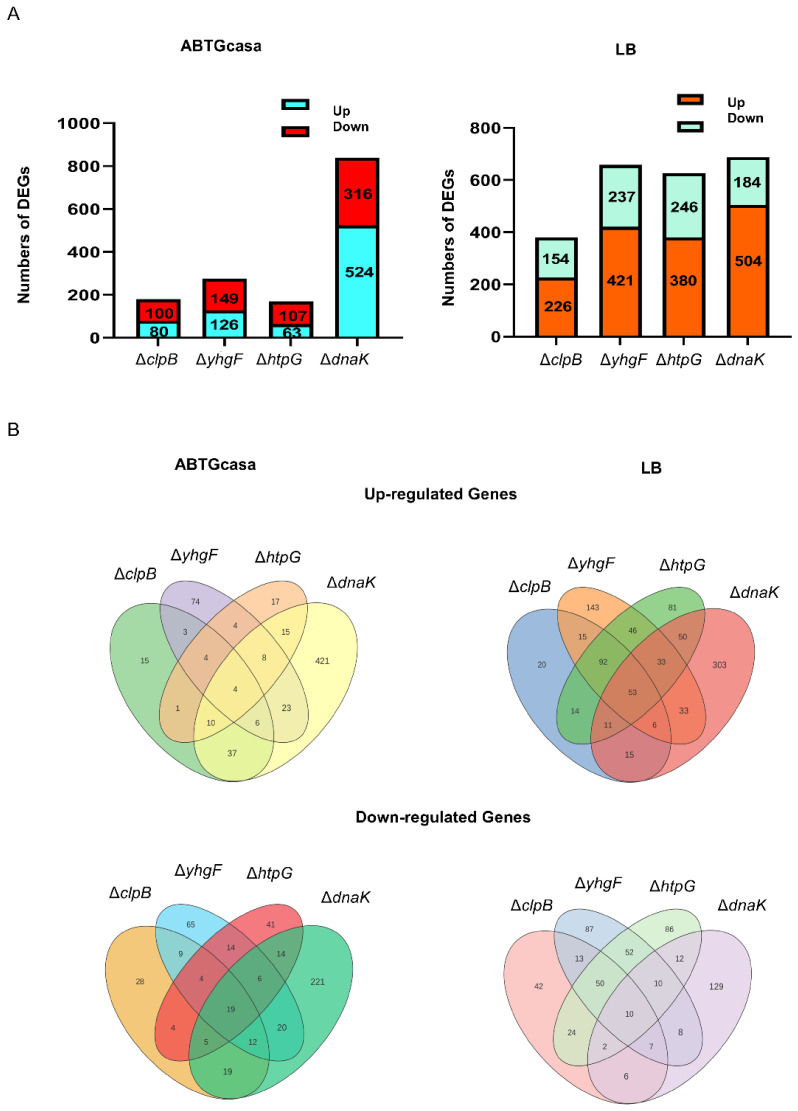
The number and Venn analysis of DEGs in Δ*clpB*, Δ*yhgF*, Δ*htpG*, and Δ*dnaK* mutants by transcriptome analysis. (**A**) The number of DEGs are shown in histogram for each mutant exponentially grown in ABTGsasa or LB medium; the up- and down-regulated DEGs in each mutant are as indicated by different colors. The DEGs were identified by using criteria of *p* value < 0.05, |log FC| > 1. (**B**) Venn diagrams were made for the up-regulated or down-regulated DEGs within four mutant comparisons with various combinations as indicated using the ggplot2 package in R (version 3.5.0).

The RNA-seq analysis (Figure 3A) showed that: (i) the number of DEGs varied among different mutants and was contingent upon the growth medium for each mutant, indicating that the roles of ClpB, YhgF, HtpG, or DnaK in transcription are growth medium-dependent; (ii) in ABTGcasa medium, fewer than 275 DEGs were detected in Δ*clpB*, Δ*yhgF*, or Δ*htpG* cells, while 840 DEGs were identified in Δ*dnaK* cells grown, suggesting that DnaK may play an important role in transcription under this condition; (iii) in LB medium, 380 DEGs were observed in Δ*clpB* cells, and more than 600 DEGs were found in Δ*yhgF*, Δ*htpG*, or Δ*dnaK* cells. The similarity in DEG numbers among these latter three mutants suggests that YhgF, HtpG, and DnaK have a comparable impact on the transcriptome in LB.

To understand the functional similarities and differences among ClpB, YhgF, HtpG, or DnaK in transcription, we performed Venn analysis using the RNA-seq data. As shown in Figure 3B, 23 common DEGs were found in four mutants grown in ABTGcasa, and 63 common DEGs were observed in LB (Figure 3B), indicating that ClpB, YhgF, HtpG or DnaK participates in transcription regulation of the same genes. Notably, in ABTGcasa, Δ*clpB* and Δ*dnaK* cells share 112 common DEGs, while other pairs of mutants share fewer DEGs. In LB, Δ*yhgF* and Δ*htpG* cells share 346 common DEGs (Figure 3B). These results suggest that ClpB and DnaK are involved in transcription regulation of a few of the same genes in ABTGcasa, while YhgF and HtpG do the same in LB. Once again, the effects of ClpB, YhgF, HtpG, or DnaK on transcription are growth medium-dependent. It should be noted that each mutant exerted an influence on the transcription of many mutant-specific genes in both media, and Δ*dnaK* cells affected the transcription of many genes (Figure 3B).

### 3.4. The Absence of ClpB, YhgF, or HtpG Affects Transcription of Genes Associated with Mutant-Specific Cellular Processes When Cells Are Grown in ABTGcasa Medium

To elucidate the cellular processes and molecular function transcriptionally regulated by ClpB, YhgF, or HtpG, Gene Ontology (GO) analyses were performed for DEGs in Δ*clpB*, Δ*yhgF*, or Δ*htpG* cells exponentially grown in ABTGcasa and LB media. In ABTGcasa, DEGs in Δ*clpB* cells were enriched in terms related to cell adhesion, biofilm formation, DNA integration and RNA ligase (ATP) activity (Table 4, Supplementary Materials Figure S6). This suggests that the absence of Δ*clpB* may induce cellular stress by affecting transcription of genes involved in biofilm formation. DEGs of Δ*yhgF* cells were enriched in motility-related categories (Table 4), suggesting that YhgF negatively regulates flagellar biosynthesis and motility-related gene expression. DEGs of Δ*htpG* cells were enriched in GO terms including transmembrane transport, aerobic electron transport chain, regulation of transcription, and RNA biosynthesis (Table 4, Supplementary Materials Figure S6), suggesting that HtpG may mainly be involved in the control of gene transcription and RNA biosynthesis. Collectively, ClpB, YhgF, or HtpG is involved in transcription regulation of genes associated with specific cellular processes, in agreement with their growth medium-dependent interaction with RpoD observed in ABTGcasa.

When cells were cultured in LB, DEGs in Δ*clpB* cells were enriched in transport activity and carbohydrate metabolism of galactose and polysaccharide (Table 4, Supplementary Materials Figure S6). DEGs in Δ*yhgF* cells were enriched in cell motility and polysaccharide metabolism (Table 4, Supplementary Materials Figure S6). In Δ*htpG* cells, DEGs were enriched in LPS biosynthesis, transport activity, and kinase regulation, pointing to a role in substance transport and LPS metabolism (Table 4, Supplementary Materials Figure S6). Notably, DEGs from all three mutants were mostly enriched in pathways related to polysaccharide metabolism and transport activities, which are required for essential cellular activities. This implies that the effect of ClpB, YhgF or HtpG on gene transcription might have a general impact on the LB growth condition.

### 3.5. Amino Acid or Lipopolysaccharide Biosynthesis Is Down-Regulated in ΔclpB, ΔyhgF, and ΔhtpG Cells in a Growth Medium-Dependent Manner

**Figure 4:**
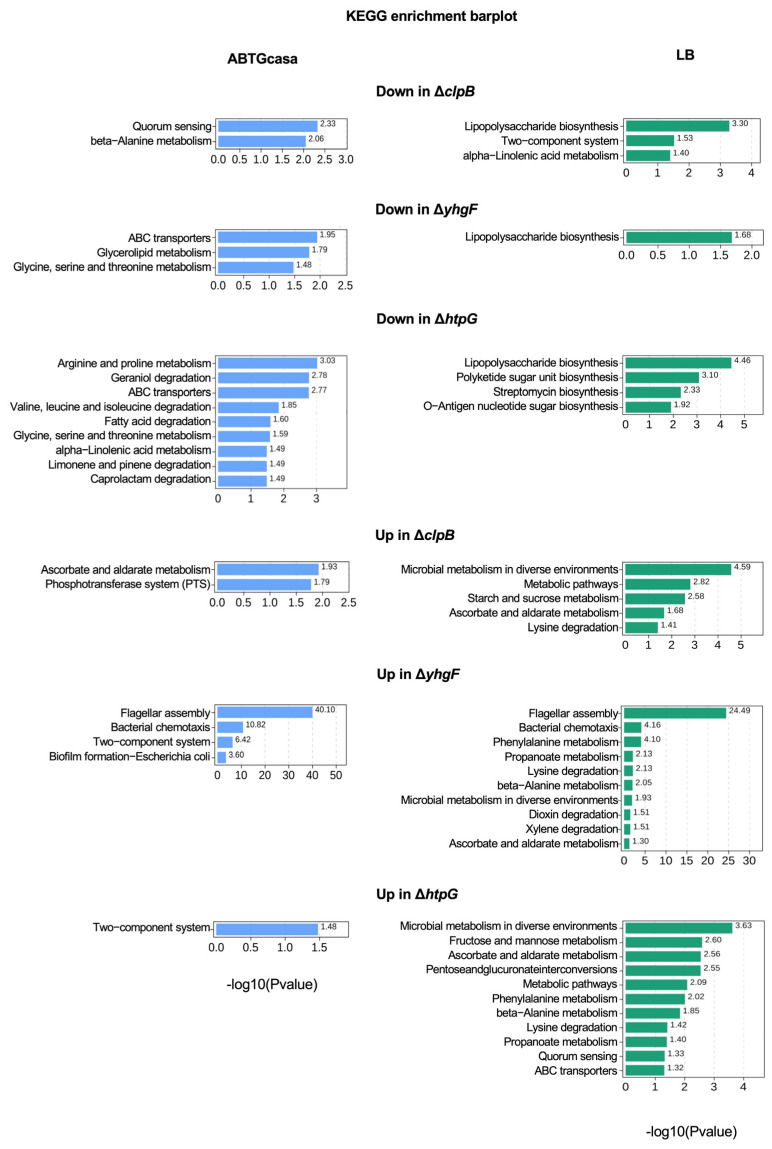
KEGG pathway enrichment analysis of DEGs in Δ*clpB*, Δ*yhgF*, and Δ*htpG* mutants. The growth conditions and data collection were as mentioned in the legend to Figure 3. The DEGs were identified using the criteria of *p* value < 0.05, |log FC| > 1. The cellular processes associated with the up- or down-regulated DEGs in each mutant under each growth condition are indicated.

To further elucidate the key biochemical metabolic and signal transduction pathways transcriptionally influenced by ClpB, YhgF, or HtpG, Kyoto Encyclopedia of Genes and Genomes (KEGG) pathway analyses were performed for DEGs in Δ*clpB*, Δ*yhgF*, or Δ*htpG* cells exponentially grown in ABTGcasa and LB media (Figure 4). In ABTGcasa, down-regulated DEGs in Δ*clpB* mutant were enriched in quorum sensing and beta-alanine metabolism (Figure 4). Down-regulated DEGs in Δ*yhgF* mutant were enriched in ABC transporters, glycerolipid metabolism, and the glycine/serine/threonine metabolism pathway (Figure 4). Down-regulated DEGs in Δ*htpG* mutant were enriched in amino acid metabolism pathways, fatty acid degradation, ABC transporters, and terpenoid metabolism (Figure 4). Collectively, DEGs associated with amino acid metabolism are down-regulated in the Δ*clpB*, Δ*yhgF*, and *ΔhtpG* mutants, indicating reduced amino acid metabolism under the ABTGcasa conditions. This finding is consistent with evidence that amino acid misincorporation induces compensatory up-regulation of chaperones such as DnaK, HtpG, and ClpB [68]. Thus, we conclude that ClpB, YhgF and HtpG may be essentially involved in positive regulation of amino acid metabolism in this growth condition.

Up-regulated DEGs in Δ*yhgF* mutant were enriched in flagellar assembly, chemotaxis, two-component system (TCS), and biofilm formation (Figure 4). Up-regulated DEGs in Δ*htpG* mutant were enriched only in TCS (Figure 4). Meanwhile, up-regulated DEGs in Δ*clpB* mutant were enriched in ascorbate and aldarate metabolism and the phosphotransferase system (Figure 4). Notably, TCS-related genes—critical for environmental sensing—were up-regulated in Δ*htpG* and Δ*yhgF* but down-regulated in Δ*clpB* [69].

When the cells were grown in LB medium, down-regulated DEGs in Δ*clpB* cells were enriched in LPS biosynthesis, TCS, and alpha-linolenic acid metabolism (Figure 4). Down-regulated DEGs in Δ*yhgF* cells were enriched only in LPS biosynthesis (Figure 4). Down-regulated DEGs in Δ*htpG* cells were enriched in LPS biosynthesis and related sugar metabolism pathways (Figure 4). Across all three mutants, LPS biosynthesis genes were consistently down-regulated. LPS is a critical component of the outer membrane, contributing to bacterial survival in harsh environments and providing intrinsic resistance to many antibiotics [70]. These results suggest that, under the LB growth condition, ClpB, YhgF, and HtpG act as positive regulators of LPS biosynthesis.

Up-regulated DEGs in Δ*clpB* were enriched in starch and sucrose metabolism, ascorbate and aldarate metabolism, and the lysine degradation pathway (Figure 4). Up-regulated DEGs in Δ*htpG* were enriched in carbohydrate metabolism, amino acid metabolism, quorum sensing, and ABC transporters (Figure 4). Up-regulated DEGs in Δ*yhgF* were enriched in flagellar assembly, chemotaxis, and amino acid metabolism (Figure 4). Notably, lysine degradation emerged as a common pathway with up-regulated genes in all three mutants under the LB condition. This contrasts with the down-regulation of amino acid metabolism genes observed in these mutants when grown in ABTGcasa. This medium-dependent difference may be attributed to the fact that ClpB, YhgF, and HtpG interact with RpoD in ABTGcasa, but not in LB.

### 3.6. Amino Acid Biosynthesis Is Up-Regulated While Flagellar Assembly Is Down-Regulated in ΔdnaK Mutant

Compared to the other mutants analyzed, a more pronounced global transcriptional change was observed in Δ*dnaK* cells (Figure 3). To further investigate this, we performed GO enrichment and KEGG pathway analyses on the DEGs in Δ*dnaK* cells. In ABTGcasa medium, GO analysis found that DEGs from Δ*dnaK* cells were significantly enriched in categories related to regulation of transcription factor activity, and various stages of the transcription process; regulation of macromolecule, cellular metabolic, nucleobase-containing and nitrogen compound, and metabolic process; and cell wall organization (Table 4, Supplementary Materials Figure S7). These results suggest that DnaK deletion disrupts transcriptional regulation and broadly impairs metabolic networks, underscoring its role in gene expression and metabolic homeostasis. Associated changes in cell structure-related genes likely contribute to the Δ*dnaK* mutant’s slow growth and abnormal morphology. In contrast, in LB medium, the absence of DnaK mainly affects transcription of genes involved in cell motility (Table 4, Supplementary Materials Figure S7). The stark contrast in the functional categories enriched in Δ*dnaK* cells under different growth media demonstrates that DnaK’s influence on gene transcription is highly growth medium-dependent.

**Figure 5:**
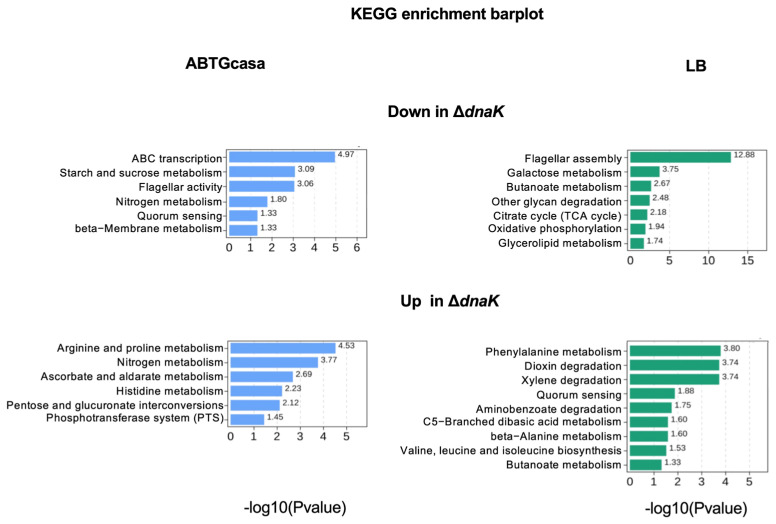
KEGG pathway enrichment analyses of DEGs in Δ*dnaK* mutant. The growth conditions and data collection were as mentioned in the legend to Figure 3. The DEGs were identified using the criteria of *p* value < 0.05, |log FC| > 1. The cellular processes associated with the up- or down-regulated DEGs in each mutant under each growth condition are indicated.

KEGG pathway analysis of DEGs from Δ*dnaK* cells revealed distinct, media-dependent enrichment patterns (Figure 5). In ABTGcasa, down-regulated DEGs of Δ*dnaK* cells were enriched in ABC transporters, carbohydrate/nitrogen metabolism, flagellar assembly, and quorum sensing (Figure 5). Up-regulated DEGs in Δ*dnaK* cells were enriched in amino acid and nitrogen metabolism, ascorbate/aldarate metabolism, pentose–glucuronate interconversions, and the phosphotransferase system (Figure 5). This metabolic reprogramming may represent a compensatory response to DnaK loss, aimed at counteracting oxidative stress, replenishing amino acids, and meeting increased energy demands.

In LB medium, down-regulated DEGs in Δ*dnaK* cells were enriched in flagellar assembly, the citrate cycle (TCA), oxidative phosphorylation, and glycerolipid/butanoate phosphorylation (Figure 5). Up-regulated DEGs in Δ*dnaK* cells were enriched in amino acid metabolism, and quorum sensing (Figure 5). These results indicate that DnaK deficiency in nutrient-rich conditions leads to repression of energy metabolism and flagellar synthesis, accompanied by activation of specific amino acid biosynthesis and stress-responsive pathways. In conclusion, despite media-dependent differences, two consistent transcriptional signatures emerged in the Δ*dnaK* mutant: (i) down-regulation of flagellar assembly genes, correlating with its reduced motility, and (ii) up-regulation of amino acid metabolism genes, suggesting a compensatory response to DnaK loss.

### 3.7. The Common DEGs Found in ΔclpB, ΔyhgF, ΔhtpG, and ΔdnaK Cells Are Associated with Amino Acid Biosynthesis

Further, we conducted KEGG and GO analyses on the common DEGs identified in Δ*clpB*, Δ*yhgF*, Δ*htpG*, and Δ*dnaK* cells. GO enrichment analysis of the common DEGs revealed distinct functional themes depending on the growth medium (Supplementary Materials Figure S7). In ABTGcasa, common DEGs were enriched in aminoacyl-tRNA ligase activity, aminoacyl-tRNA editing, aminopeptidase activity, shikimate 3-dehydrogenase (NADP^+^), and nutrient reservoir functions (Supplementary Materials Figure S7). This indicates that the loss of any of these proteins triggers a common transcriptional response centered on translation quality control and metabolic adjustment. In LB, the common DEGs were enriched in carboxylic acid catabolism, monosaccharide metabolism, base-excision repair, the spliceosomal complex, oxidoreductase activity, response to reactive oxygen species, and signal peptidase processing (Supplementary Materials Figure S7). This contrasts with the translation-focused enrichment observed in ABTGcasa, indicating that the transcriptional impact of ClpB, YhgF, HtpG, and DnaK is broader in LB but more specific in ABTGcasa. This contrast correlates with the absence of their interaction with RpoD in LB versus its presence in ABTGcasa (Figure 1B), reinforcing that direct σ factor binding channels their regulatory influence toward specific cellular functions.

In ABTGcasa medium, KEGG analysis revealed that down-regulated common DEGs were enriched in phenylalanine, tyrosine and tryptophan biosynthesis, while up-regulated common DEGs were enriched in monobactam and lysine biosynthesis (Table 5). In LB medium, the number of common down-regulated DEGs was insufficient for reliable KEGG analysis. However, up-regulated common DEGs were enriched in lysine degradation, and phenylalanine, fructose and mannose metabolism (Table 5). These findings indicate that the absence of the *clpB*, *htpG*, *dnaK*, and *yhgF* gene affects the transcription of genes involved in amino acid biosynthesis, which may help the mutants to adapt to the different growth conditions.

### 3.8. Initiation of Chromosome Replication Is Delayed in ΔdnaK Mutant with a Slower Growth

**Figure 6:**
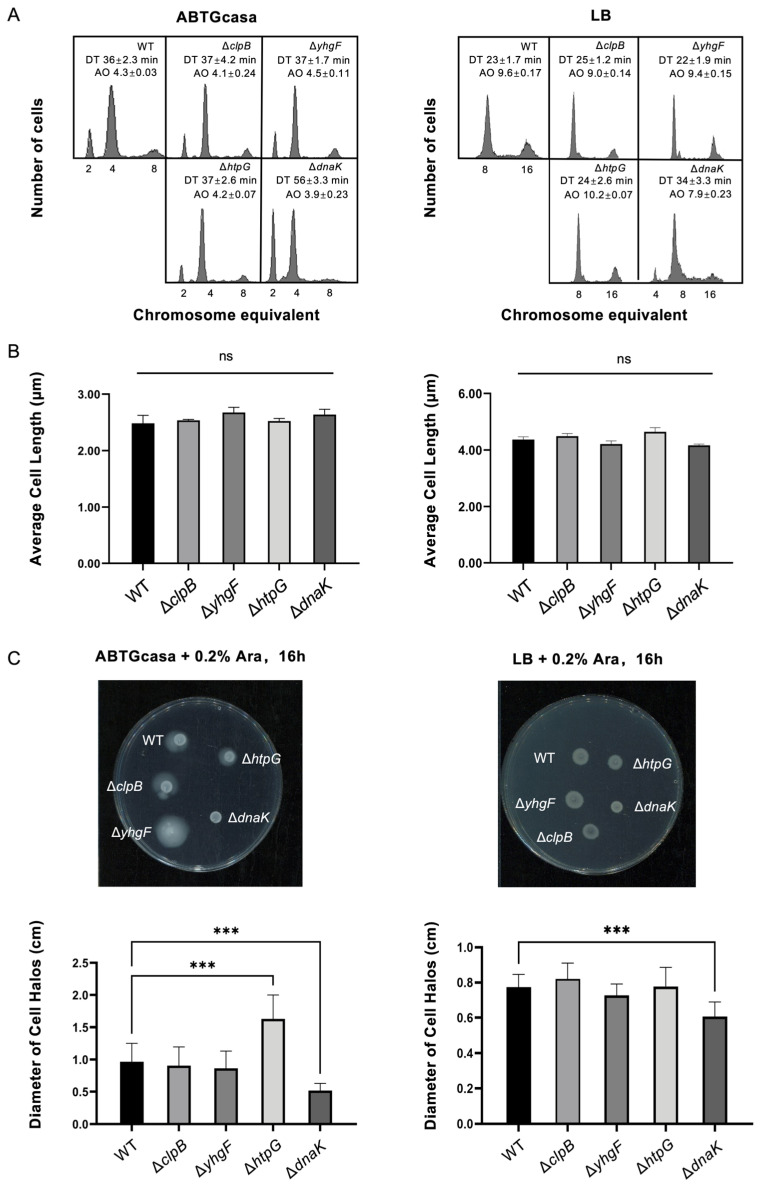
Initiation of replication is delayed in Δ*dnaK* mutant with a slow growth. (**A**) Exponentially growing cultures of WT, Δ*clpB*, Δ*yhgF*, Δ*htpG*, and Δ*dnaK* cells in ABTGcasa or LB medium were treated with rifampicin and cephalexin for 3–5 generations, thereby stopping initiation of replication and cell division while allowing ongoing replication to finish. Cells were stained with Hoechst33258 for 30 min and analyzed by flow cytometer (BD Fortesa, Franklin Lakes, NJ, USA). Chromosome equivalents per cell (X-axis) are plotted against cell number (Y-axis) for 10,000 cells. The BD FloJo^TM^ software was used to calculate the average number of origins per cell (A.O.). The doubling time and genotype of the cells are as shown. (**B**) Cell size analysis. Cell dimensions were determined by microscopy (*n* > 100 per strain). Values are the mean of three independent experiments. ns, not significant (*p* > 0.05). (**C**) Motility assay. Cell motility was measured as described in Materials and Methods. Halo diameters were measured, and the mean from at least three independent experiments is shown. All data are presented as the mean ± SD of three independent biological replicates. Statistical significance was determined using a two-tailed, paired Student’s *t*-test. *** *p* < 0.001.

To investigate the phenotypic alteration in Δ*clpB*, Δ*yhgF*, Δ*htpG*, or Δ*dnaK* mutant due to the global transcription changes, the cell cycle parameters (replication pattern, cell size, and growth rate) of these mutants were measured during the exponential growth phase. Cells were grown to an optical density at 450 or 600 nm (OD_450_ or OD_600_) of 0.15 in either ABTGcasa or LB medium, then treated with rifampicin and cephalexin for 3–5 generations to stop initiation of replication and cell division [52]. The replication pattern of the cells was subsequently analyzed by flow cytometry, as described in the Materials and Methods. In ABTGcasa medium, the highest number of WT cells contained four chromosomes, followed by cells containing either two or eight chromosomes (Figure 6A, left), which corresponded to the number of replication origins per cell. The two-chromosome cells showed that these young cells had not yet initiated replication. The eight-chromosome cells indicated that replication had initiated before the previous cell division [53]. It should be noted that the number of replication origins is equivalent to the number of chromosomes after treatment with rifampicin and cephalexin [52]. The average number of replication origins per cell (A.O.) in WT cells was 4.3, with a doubling time (DT) of 36 min. The replication pattern and DT of Δ*clpB*, Δ*htpG*, or Δ*yhgF* cells were largely similar to those of WT cells (Figure 6A, left). However, in Δ*dnaK* cells, the number of cells with two chromosomes was increased, while the number of cells with eight chromosomes was decreased, and A.O. was 3.9 with DT of 56 min (Figure 6A, left). This indicates that initiation of chromosome replication is delayed with slower growth. In LB medium, WT cells had A.O. of 9.6 with DT of 23 min, whereas Δ*dnaK* cells contained A.O. of 7.9 with DT of 34 min (Figure 6A, right), also showing a delayed initiation and slow growth in Δ*dnaK* cells. Again, the replication pattern and DT of Δ*clpB*, Δ*htpG*, or Δ*yhgF* cells were like those in WT cells (Figure 6A, right). These results suggest that the absence of ClpB, HtpG, or YhgF does not affect the cell cycle progression, but the lack of DnaK leads to a delay in initiation with slow growth in both ABTGcasa and LB. Further, microscopic analysis revealed no significant differences in cell size between any of the mutants and WT cells in either growth medium (Figure 6B).

As mentioned above, transcription of genes associated with flagellar assembly was down-regulated in Δ*dnaK* cells (Figure 5), but up-regulated in Δ*yhgF* cells grown in both ABTGcasa and LB (Figure 4). To determine whether these transcriptional changes translate into phenotypic differences, we assessed the motility of each mutant on semi-solid agar plates supplemented with arabinose, as previously described [71]. Consistent with the down-regulation of motility-related genes (Figure 5), diameters of cell halos of Δ*dnaK* mutant grown in both LB (0.6 cm) and ABTGcasa (0.5 cm) were smaller than those of WT cells (0.8 cm in LB and 1 cm in ABTGcasa) (Figure 6C). In contrast, the cell halo diameter of Δ*yhgF* mutant (1.6 cm) grown on ABTGcasa was bigger than that of WT cells (1 cm), correlating with the up-regulation of the cell motility genes in the mutant (Figure 4). However, any significant increase in cell halo diameter was not observed in Δ*yhgF* mutant grown on LB, although an increase in transcription of cell motility genes was found (Figure 4). This may be explained by the absence of the YhgF interaction with RpoD in LB (Figure 1), suggesting that YhgF’s influence on motility is not solely dependent on transcriptional regulation but may require its physical association with the σ factor.

## 4. Discussion

### 4.1. The Molecular Chaperones Interacting with the σ^70^ Factor Are Involved in Transcription Control

As shown in Figure 2, the molecular chaperones ClpB, DnaK, HtpG, and the RNA-binding protein YhgF, co-localize with the nucleoid as the σ^70^ factor (RpoD) does. This suggests that these proteins might associate with the nucleoid, and potentially contribute to its remodeling [61]. In turn, such chaperone-mediated nucleoid remodeling could influence transcription [61] and/or DNA replication. Consistent with these findings, the CbpA, CbpB, DnaA, Dps, Fis, Hfq, H-NS, HU, IciA, IHF, Lrp and StpA proteins are associated with the nucleoid in *E. coli* [72], playing regulatory roles in DNA replication and/or transcription of the growth-related genes [72]. Indeed, the nucleoid proteins H-NS, IHF and DnaA are involved in nucleoid organization, replication initiation and cell division [73]. The *E. coli* TorR, acting as a transcription factor, co-localizes with the nucleoid in a cell cycle-dependent fashion [45]. The *Clostridium difficile* HupA protein, a homolog of the *E. coli* HU, also co-localizes with the nucleoid [74]. In eukaryotic cells, chaperones are also known to mediate nucleosome assembly [63].

The σ^70^ factor plays a central role in transcription by associating with RNAP, recognizing promoter sequences, and guiding RNAP assembly at promoters [19]. Its activity can be modulated by regulatory proteins; for instance, the AisA protein, encoded by the bacteriophage T4, binds to and remodels the σ^70^ factor, thereby inhibiting its normal contact with promoter DNA [30]. Here, we observed that the ClpB, DnaK, HtpG chaperones and YhgF interact with the σ^70^ factor both in vivo and in vitro; the interaction in vivo is in a growth medium-dependent fashion (Figure 1). The protein–protein interaction of ClpB, DnaK, HtpG and YhgF with the σ^70^ factor further suggests that these proteins might be involved in regulation of gene transcription. Indeed, as presented in Figure 3, more than a hundred DEGs in Δ*clpB*, Δ*dnaK*, Δ*htpG*, or Δ*yhgF* mutant grown in ABTGcasa medium are identified by transcriptome analysis, while more than 300 DEGs are observed in each mutant grown in LB. The numbers of DEGs for mutants are different and growth medium-dependent.

Further, GO enrichment analysis (Table 4, Supplementary Materials Figure S6) shows that (i) the DEGs of Δ*clpB* mutant were associated with biofilm formation in ABTGcasa, while they were related with carbohydrate metabolism in LB; (ii) the DEGs of Δ*yhgF* mutant were consistently enriched in the cell motility process in both media; (iii) the DEGs in Δ*htpG* cells were linked to RNA and macromolecule metabolism in ABTGcasa, but to transporter activity in LB. These findings demonstrate that the transcriptional roles of ClpB, YhgF, and HtpG are both protein- and environment-specific. Notably, the observed alterations in cell motility and biofilm formation, a known stress-induced response [75], may represent adaptive strategies enabling bacterial cells to cope with changing intra- and extracellular environments. KEGG pathway analysis further highlighted medium-dependent metabolic reprogramming. In ABTGcasa, DEGs enriched in amino acid metabolism were down-regulated in all Δ*clpB*, Δ*yhgF*, and Δ*htpG* mutants. In LB, DEGs enriched in LPS biosynthesis were down-regulated across these mutants (Figure 4). These results are consistent with a previous study, in which multiple transcriptional factors regulate transcription of the *rpoE* gene, encoding for the RpoE σ factor, under different growth conditions and when the LPS biosynthesis is defective [76]. Interestingly, in LB medium, DEGs enriched in the lysine degradation process were up-regulated in all the mutants (Figure 4). It is likely that the up-regulated lysine degradation and the down-regulated amino acid metabolism would maintain a certain level of amino acid concentration, which may be required for normal cellular physiological condition.

**Figure 7:**
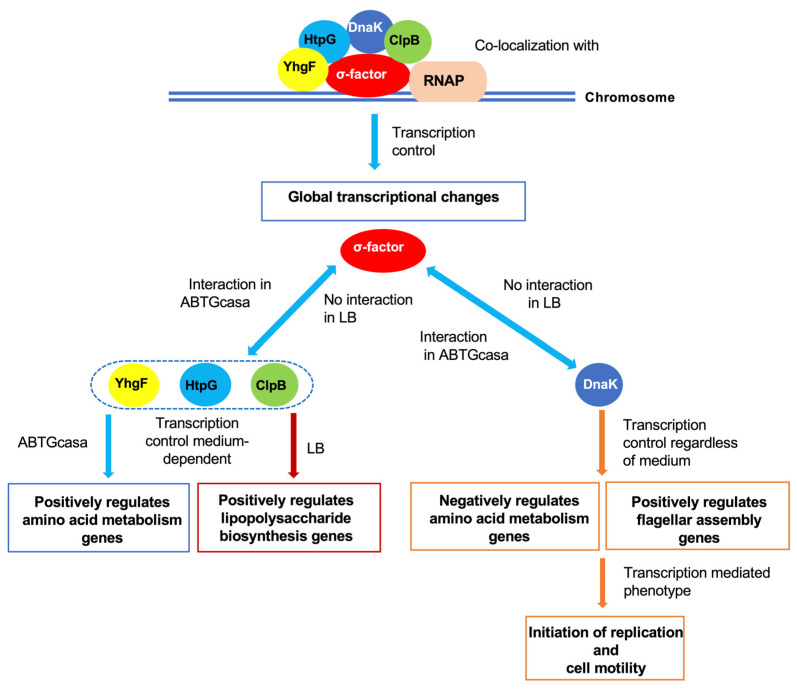
Schematic summary of the main findings. The σ^70^ factor, along with ClpB, YhgF, HtpG, and DnaK, co-localizes with the nucleoid. By interacting with σ^70^, these proteins influence the transcription of genes involved in amino acid metabolism, lipopolysaccharide biosynthesis, and flagellar assembly. Interactions of the σ^70^ factor with ClpB, YhgF, or HtpG, and their subsequent effects on transcription, occur in a growth medium-dependent manner (blue arrows). Independently of their interaction with σ^70^, the molecular chaperones also regulate the expression of genes involved in specific cellular processes, as indicated (red arrows). DnaK regulates gene expression through both σ^70^-dependent and -independent mechanisms, impacting distinct cellular processes under specific growth conditions (orange arrows).

As summarized in Figure 7, the findings in this work indicate that the ClpB, YhgF, and HtpG proteins positively regulate amino acid metabolism and LPS biosynthesis in a growth medium-dependent manner, consistent with their medium-dependent interaction with the σ^70^ factor in vivo (Figure 1). Thus, it is reasonable to hypothesize that the protein–protein interaction of the molecular chaperones with the σ^70^ factor could mediate dynamic recycling of the σ^70^ factor in RNAP assembly at the promoter. In other words, the molecular chaperones mediate association of the σ^70^ factor with RNAP, and RNAP assembly at a chosen promoter, and subsequent release of the factor from the RNAP complex after transcription initiation. Then, the released σ^70^ factor leads a new round of RNAP assembly and transcription at the next promoter. Such a dynamic recycling of the σ^70^ factor guides global transcription to select genes for transcription and subsequently allows cells to cope with the changing environments. In the chaperone-mediated recycling of the σ^70^ factor, nutrient levels or specific components in growth media likely function as a signal for the recycling since growth medium-dependent transcription (Figure 3, Figure 4, Figure 5 and Figure 7) results from the growth medium-dependent interaction of the chaperones with the σ^70^ factor (Figure 1). As supporting evidence for the proposal mentioned above, the AisA protein remodels the structure of the σ^70^ factor, inhibiting its normal contact with the promoter sequence [30]. These examples illustrate that protein–protein interactions can modulate σ factor function and RNAP dynamics, lending credence to our proposed hypothesis. Similarly, the DksA protein peels off the stalled RNAP through protein–protein interaction at damaged sites on the DNA template [77] and a Swi2/Snf2 protein also recycles RNAP during transcription [31]. Generally, molecular chaperones interact and protect substrate proteins by preventing protein misfolding and are adaptable dynamic proteins under unfavorable conditions [20,21]. Our findings extend this paradigm by implicating chaperones in the active regulation of transcription through direct interaction with a key component of the transcriptional machinery.

### 4.2. The DnaK Chaperone Facilitates Initiation of Replication and Cell Motility Through Transcription

As depicted in Figure 6, the absence of DnaK leads to a delayed initiation of chromosome replication, slow growth, and a decreased cell motility regardless of the growth medium. The delayed replication initiation of Δ*dnaK* mutant may be linked to the up-regulation of DNA repair genes, including *dinB*, *dnaQ*, *umuD*, and *polB* (Supplementary Materials Table S5). The polymerases encoded by these genes are involved in DNA damage repair, a process known to halt replication initiation [78]. This interpretation is consistent with the established role of DnaK in DNA damage responses [79], and the involvement of the ClpB/DnaK/DnaJ/GrpE chaperone system in activating DNA replication factors [80,81]. Alternatively, the replication defect could be an indirect consequence of the extensive transcriptional reprogramming observed in the Δ*dnaK* mutant (over 600 DEGs, Figure 3), which may broadly perturb cell cycle progression. The slow growth phenotype of the mutant (Figure 6) supports this view, as reduced growth rates are often associated with delays in cell cycle progression [54]. The absence of QseB/QseC two-component signaling leads to an early initiation of chromosomal replication and higher concentration of DnaA [71]. Therefore, although any difference in expression of DnaA was not observed in Δ*dnaK* cells, a change in DnaA expression should directly regulate replication initiation, given that DnaA is an initiator for replication and also a transcription factor [82]. Recently, a DnaA-dependent riboswitch was reported to attenuate the transcription of the *his* operon [83]. The decreased cell motility of Δ*dnaK* cells (Figure 6C) can be directly attributed to the down-regulation of motility-related genes observed in our transcriptomic analysis (Figure 5), a finding consistent with previous reports [71]. Mechanistically, DnaK has been shown to interact with QseB, a regulator of motility, and with FtsZ, a key cell division protein; disruption of these interactions leads to decreased motility [71]. Taken together, these findings support a model (Figure 7) in which DnaK integrates multiple cellular processes—including DNA replication, cell cycle progression, and motility—through both direct protein–protein interactions and indirect transcriptional effects, enabling cells to adapt to environmental changes.

### 4.3. Clinical Implications of Chaperone-σ^70^ Interactions in E. coli Pathogenesis

GO and KEGG analyses revealed that the DEGs in chaperone mutants are enriched in pathways directly relevant to bacterial pathogenesis, including LPS biosynthesis, flagellar assembly, biofilm formation, and amino acid metabolism. LPS is a major determinant of *E. coli* pathogenicity, triggering host inflammatory responses [84]. Dysregulation of LPS biosynthesis in chaperone mutants (Figure 4) may thus alter the pathogen’s immunostimulatory properties and susceptibility to host defenses. Flagellar assembly is similarly essential for motility and chemotaxis, enabling the pathogen to reach favorable niches and establish infection [85]. Biofilm formation, another enriched pathway, is a well- established driver of chronic and recurrent *E. coli* infections, particularly those associated with urinary catheters and indwelling devices [86]. Together, these observations underscore the clinical relevance of the chaperone-σ^70^ axis.

The extensive transcriptional reprogramming observed in the Δ*dnaK* mutant (over 600 DEGs, Figure 3), along with its weakness in replication initiation and growth, suggests that DnaK may be a potential antimicrobial target. Targeting chaperone networks sensitizes multidrug-resistant *E. coli* to antibiotics [87], implying that pharmacological inhibition of DnaK or its interaction with σ^70^ could be a potential antibiotic. Analogously, targeting Hsp70 overcomes chemoresistance in cancer by blocking its protective role against proteotoxic stress and apoptosis [88,89], suggesting that chaperone-targeting approaches may represent a broadly applicable therapeutic principle for drug-resistant diseases.

The protein–protein interactions characterized between σ^70^ and ClpB, HtpG, YhgF, or DnaK represent potential targets for small-molecule inhibitors. Molecular docking demonstrated that RpoD forms hydrogen bond networks with ClpB (25 residues), DnaK (27 residues), HtpG (19 residues), and YhgF (20 residues), which provides a structural basis for their specific interactions (Supplementary Materials Figures S10–S13). Disrupting these interactions could selectively impair σ^70^-dependent transcription of virulence genes without affecting housekeeping gene expression, offering a novel anti-virulence approach. Unlike traditional antibiotics, anti-virulence strategies exert weaker selective pressure for resistance [90], making them an appealing direction for future therapeutic development.

In summary, our findings establish a mechanistic link between chaperone-mediated regulation of σ^70^ recycling and subsequent key bacterial processes. It could be proposed that the chaperone–σ^70^ interaction network represents both a fundamental transcriptional control mechanism and a promising target for innovative antimicrobial strategies against *E. coli* infections.

## Figures and Tables

**Table 1:** Bacterial strains used.

Strain	Genotype	Reference or Source
BW25113	Wild-type rrnB3Δ*lacZ*4787 *hsdR*514Δ (*araBAD*) 567Δ (*rhaBAD*)	[42]
DH5α	F^-^ *sup*E44Δ*lac*U169 (Δ*lacZ*Δ*M*15) *hsdR*17 *recA*1 *end A*1 gyrA96	New England Biolabs (Ipswich, MA, USA)
BL21(DE3)	*E. coli* B F^-^*ompT* *hsdS*B (r_B_^-^m_B_^-^) *dcm*^+^ Tet^r^ *gal* (DE3) *endA* Hte	Agilent Technologies
BTH101	F *cya*-99 *araD*139 *galE*15 *galK*16 *rpsL*1(*Strr*) *hsdR*2 *mcrA*1 *mcrB*1	[43]
Δ*danK*	BW25113Δ*danK*:: *neo* (*kan^R^*)	This work
Δ*clpB*	BW25113Δ*clpB:*:*neo* (*kan^R^*)	This work
Δ*htpG*	BW25113Δ*htpG:*:*neo* (*kan^R^*)	This work
Δ*yhgF*	BW25113Δ*yhgF:*:*neo* (*kan^R^*)	This work
pCA24N-*rpoD*/BW25113	BW25113/pCA24N-*rpoD*	This work
pCA24N-*rpoD*/Δ*clpB*	BW25113Δ*clpB:*:*neo* (*kan^R^*)/pCA24N-*rpoD*	This work
pCA24N-*rpoD*/Δ*htpG*	BW25113Δ*htpG:*:*neo* (*kan^R^*)/pCA24N-*rpoD*	This work
pCA24N-*rpoD*/Δ*yhgF*	BW25113Δ*yhgF:*:*neo* (*kan^R^*)/pCA24N-*rpoD*	This work
pCA24N-*rpoD*/Δ*dnaK*	BW25113Δ*dnaK:*:*neo* (*kan^R^*)/pCA24N-*rpoD*	This work
pCA24N-*clpB*/Δ*clpB*	BW25113Δ*clpB:*:*neo* (*kan^R^*)/pCA24N-*clpB*	This work
pCA24N-*htpG*/Δ*htpG*	BW25113Δ*htpG:*:*neo* (*kan^R^*)/pCA24N-*htpG*	This work
pCA24N-*yhgF*/Δ*yhgF*	BW25113Δ*yhgF:*:*neo* (*kan^R^*)/pCA24N-*yhgF*	This work
pCA24N-*dnaK*/Δ*dnaK*	BW25113Δ*dnaK:*:*neo* (*kan^R^*)/pCA24N-*dnaK*	This work
pET28a-*clpB*/BL21(DE3)	BL21(DE3)/pET28a-*clpB*	This work
pET28a-*rpoD*/BL21(DE3)	BL21(DE3)/pET28a-*rpoD*	This work
pET28a-*yhgF*/BL21(DE3)	BL21(DE3)/pET28a-*yhgF*	This work
pET28a-*htpG*/BL21(DE3)	BL21(DE3)/pET28a-*htpG*	This work
pET28a-*dnaK*/BL21(DE3)	BL21(DE3)/pET28a-*dnaK*	This work

**Table 2:** The known biological functions of RpoD, ClpB, HtpG, YhgF, and DnaK in *E. coli* (https://www.uniprot.org, accessed on 20 May 2026).

Gene	Protein	Key Biological Roles	Entry
***rpoD***	RNA polymerase sigma factor RpoD (σ^70^)	To recognize promoter sequences; to initiate housekeeping gene transcription	P63284
***clpB***	Hsp100 chaperone protein ClpB	To reactivate aggregated proteins; thermotolerance	P63284
***htpG***	Hsp90 chaperone protein HtpG	To associate with protein maturation; heat shock adaptation	P0A6Z3
***yhgF***	RNA-binding protein YhgF	To modulate transcription elongation; to interact with RNA polymerase; to function in DNA repair and recombination [58]	P46837
***dnaK***	Hsp70 chaperone protein DnaK	To associate with protein folding; replication initiation; stress response	P0A6Y8

**Table 3:** Binding kinetic parameters of RpoD with ClpB, HtpG, YhgF, or DnaK in SPR analysis.

Protein	*K_a_* (1/Ms)	*K_d_* (1/s)	*K_D_* (M)
ClpB	1.405 × 10^3^	2.347 × 10^−4^	1.671 × 10^−7^
HtpG	5.886 × 10^3^	1.108 × 10^−3^	1.882 × 10^−7^
YhgF	1.215 × 10^4^	4.629 × 10^−4^	3.809 × 10^−8^
DnaK	3.221 × 10^4^	3.440 × 10^−4^	1.056 × 10^−8^

Proteins were purified from cells grown in LB medium at 37 °C. RpoD (σ^70^ factor) was immobilized on a CM5 sensor chip with various concentrations, SPR analysis was performed as mentioned in Materials and Methods. The experiment was repeated three times.

**Table 4:** Summary of GO enrichment analysis of DEGs in Δ*clpB*, Δ*yhgF*, Δ*htpG*, and Δ*dnaK* mutants.

ABTGcasa	LB
**Δ*clpB***	
cell adhesion	amide/peptide/nitrogen compound transport
biofilm formation	galactosyltransferase activity
DNA integration	cellular polysaccharide biosynthetic/metabolic process
RNA ligase (ATP) activity	oxidoreductase activity
-	membrane-enclosed/organelle lumen
**Δ*yhgF***	
cell motility	flagellum-dependent cell motility,
locomotion	localization
flagellum assembly	amide/peptide/nitrogen compound transport
chemotaxis	cellular polysaccharide
**Δ*htpG***	
transmembrane transport	flavin adenine dinucleotide/FAD binding
aerobic electron transport chain	lipopolysaccharide biosynthetic/metabolism process
regulation of transcription	oligopeptide/p-aminobenzoyl-glutamate/dipeptide/modified amino acid transmembrane transporter activity
RNA biosynthesis	potassium ion transport
-	kinase regulator activity
**Δ*dnaK***	
nucleic acid binding transcription factor/transcription factor activity	pilus/pilus assembly
regulation of transcription/RNA biosynthesis/cellular metabolic/macromolecule metabolic process	cell/biological adhesion
cell wall organization	cell projection
-	flagellum-dependent cell motility
-	cell localization

The growth conditions and data collection are as mentioned in the legend to Figure 3. The DEGs were identified by using criteria of *p* value < 0.05, |log FC| > 1. The cellular processes in which the DEGs are involved, and the genotype of the cells, are as indicated. The detailed analysis is presented in the Supplementary Materials Figures S5 and S6.

**Table 5:** KEGG pathway enrichment analyses of the common DEGs found in all Δ*clpB*, Δ*yhgF*, Δ*htpG*, and Δ*dnaK* mutants. The growth conditions and data collection were as mentioned in the legend to Figure 3.

ABTGcasa	LB
**Down**	
Phenylalanine, tyrosine and tryptophan biosynthesis	-
**Up**	
Monobactam biosynthesis	Microbial metabolism in diverse environments
Lysine biosynthesis	Phenylalanine metabolism
-	Lysine degradation
-	Fructose and mannose metabolism

## Data Availability

The raw sequencing data have been deposited in the Sequence Read Archive (SRA) and are accessible under BioProject accession PRJNA1338492. For the purpose of peer review, the data can be accessed via the following reviewer link: https://dataview.ncbi.nlm.nih.gov/object/PRJNA1338492?reviewer=g305h5ou3qibrndusqe9nsmh0e (accessed on 25 May 2026).

## Supplementary Materials

The following supporting information can be downloaded at: https://www.mdpi.com/article/10.3390/genes17060621/s1, Figure S1: Characterizations of *ΔclpB*, *ΔyhgF*, *ΔhtpG*, and *ΔdnaK* mutants by PCR technique. To confirm the correctness in construction of BW25113*ΔclpB::neo (kanR)*; Figure S2: Co-immunoprecipitation with RpoD and protein purification; Figure S3: No co-localization of GFP empty vector (pCA24N) with the nucleoids in BW25113; Figure S4: RpoD, ClpB, YhgF, HtpG, and DnaK co-localize with nucleoids; Figure S5: Co-localization of RpoD with the nucleoids in ΔclpB (A), ΔyhgF (B), ΔhtpG (C), and ΔdnaK (D) mutants in ABTGcasa; Figure S6: GO enrichment analysis of DEGs in ΔclpB, ΔyhgF, and ΔhtpG mutants; Figure S7: GO pathway enrichment analyses of DEGs in ΔdnaK mutant and the common DEGs found in ΔclpB, ΔyhgF, ΔhtpG, and ΔdnaK mutants; Figure S8: Relative RNA levels of the chosen DEGs in ΔclpB, ΔyhgF, ΔhtpG, or ΔdnaK mutants; Figure S9: Relative RNA levels of the common DEGs in ΔclpB, ΔyhgF, ΔhtpG, and ΔdnaK mutants; Figure S10: Molecular interactions between RpoD and ClpB. RpoD is shown in green and ClpB in purple; Figure S11: Molecular interactions between RpoD and HtpG. RpoD is shown in green and HtpG in Orange; Figure S12: Molecular interactions between RpoD and YhgF. RpoD is shown in green and YhgF in blue; Figure S13: Molecular interactions between RpoD and DnaK; Table S1: Plasmids used; Table S2: Primers used; Table S3: Primers 3 used in RT-qPCR analysis; Table S4: The proteins pulled-down with 4 RpoD in RpoD-GFP-Trap immunoprecipitation; Table S5: The 4 DEGs associated with DNA replication 6 in *ΔdnaK* mutant; Table S6: The components of 1 L ABTGcasa and LB medium; Table S7: Sequencing data statistics.
