# Supplementary material for: The Roles of Molecular Chaperones Interacting with the σ70 Factor in Global Transcription of the Escherichia coli Genome
Source: Genes (Basel). 2026 May 29;17(6):621. doi: 10.3390/genes17060621 (PMC13298495; doi:10.3390/genes17060621)
Supplement: Supplementary file 1 [file genes-17-00621-s001.zip › Supplemental Tables.pdf]

Supplementary Table S1 Plasmids used.

Plasmid	Description	Reference or source
pET28a	rep_{ColE1} *neo lacI* P_{T7}	EMD Biosciences
pKD4	Rep_{R6K} *bla* FRT *neo* FRT	[34]
pKD46	$rep_{\mathrm{pSC101}}{}^{ts}$*bla* P_{araBAD} γβ exo	[34]
pCP20	$rep_{\mathrm{pSC101}}{}^{ts}$*bla cat cI*857$P_R$	[34]
pKNT25	rep_{p15A} *neo* P_{lac}T25 (pSU19 derivative)	[38]
pUT18	rep_{ColE1} $A_P{}^R$ P_{lac}T18 (pSU19 derivative)	[38]
pUT18-*dnaK*	The *dnaK* gene was inserted into pUT18 at *Pst*I and *Kpn*I sites.	This work
pUT18-*rpoD*	The *rpoD* gene was inserted into pUT18 at *Hind*III and *Kpn*I sites.	This work
pUT18-*htpG*	The *htpG* gene was inserted into pUT18 at *Hind*III and *Kpn*I sites.	This work
pUT18-*clpB*	The *clpB* gene was inserted into pUT18 at *Hind*III and *Kpn*I sites.	This work
pUT18-*yhgF*	The *yhgF* gene was inserted into pUT18 at *Hind*III and *Kpn*I sites.	This work
pKNT25-*dnaK*	The *dnaK* gene was inserted into pKNT25 at *Pst*I and *Kpn*I sites.	This work
pKNT25-*rpoD*	The *rpoD* gene was inserted into pKNT25 at *Hind*III and *Kpn*I sites.	This work
pKNT25-*clpB*	The *clpB* gene was inserted into pKNT25 at *Hind*III and *Kpn*I sites.	This work
pKNT25-*yhgF*	The *yhgF* gene was inserted into pKNT25 at *Hind*III and *Kpn*I sites.	This work
pKNT25-*htpG*	The *htpG* gene was inserted into pKNT25 at *Hind*III and *Kpn*I sites.	This work
pCA24N-*clpB*	The *clpB* gene was inserted into pCA24N at *sfi*I site to produce *clpB*-GFP fusion.	This work
pCA24N-*rpoD*	The *rpoD* gene was inserted into pCA24N at *sfi*I site to produce *rpoD*-GFP fusion.	This work
pCA24N-*yhgF*	The *yhgF* gene was inserted into pCA24N at *sfi*I site to produce *yhgF*-GFP fusion.	This work
pCA24N-*htpG*	The *htpG* gene was inserted into pCA24N at *sfi*I site to produce *htpG*-GFP fusion.	This work
pCA24N-*dnaK*	The *dnaK* gene was inserted into pCA24N at *sfi*I site to produce *dnaK*-GFP fusion.	This work
pCA24N-*uvrY*	The *uvrY* gene was inserted into pCA24N at *sfi*I site to produce *uvrY*-GFP fusion.	This work
pET28a-*clpB*	The *clpB* gene was inserted into pET28a at *Hind*III and *BamH*I sites.	This work

pET28a-*rpoD*	The *rpoD* gene was inserted into pET28a at *Hind*III and *BamH*I sites.	This work
pET28a-*yhgF*	The *yhgF* gene was inserted into pET28a at *Hind*III and *BamH*I sites.	This work
pET28a-*htpG*	The *htpG* gene was inserted into pET28a at *Hind*III and *BamH*I sites.	This work
pET28a-*dnaK*	The *dnaK* gene was inserted into pET28a at *Hind*III and *BamH*I sites.	This work

Supplementary Table S2 Primers used.

ID No.	Sequence（5′---3′）	Usage
1	GAAACTTCTTGTCACCCGTGGTAAGGAGC	To construct the T14G mutation in *rpoD* on pCA24N-*rpoD* （ G14T mutation）
2	CGGGTGACAAGAAGTTTCAGCTGTGACTG	
3	TTATCTAGAAAATGCGTCTGGATCGTCT	To construct plasmid pUT18-*clpB* and pKNT25-*clpB*
4	TAAGGTACCCGCTGGACGGCGACAATCCGG	
5	CCGTCTAGAAAATGGAGCAAAACCCGCAG	To construct plasmid pUT18-*rpoD* and pKNT25-*rpoD*
6	CTAGGTACCGCATCGTCCAGGAAGCTACGCAGC	
7	CGCGTCTAGAAAATGATGAATGATTCGTTC	To construct plasmid pUT18-*yhgF* and pKNT25-*yhgF*
8	CGCGGTACCCGACGTTTTTTGCCCATTGCCGCC	
9	CCCAAGCTTGATGAAAGGACAAGAAACTCGTGGTT	To construct plasmid pUT18-*htpG* and pKNT25-*htpG*
10	CGCGGTACCCGTTTTGCAGTCACAACCTG	
11	CGCCGTCTAGAAAATGGGTAAAATAATTGG	To construct plasmid pUT18-*dnaK* and pKNT25-*dnaK*
12	CCGCGGATCCTCTTTTTTGTCTTTGACTTCTTCA	
13	CAGCCATATGATGCGTCTGGATCGTCT	To construct plasmid pET28a-*clpB*
14	GGGAAGCTTGCTCGTTTTATCATTTACT	
15	CAGCCATATGATGGAGCAAAACCCGCAG	To construct plasmid pET28a-*rpoD*
16	TATAAGCTTTGCCGGGTGCGGCGTAAC	
17	ATACCATGGGCATGATGAATGATTCGTTC	To construct plasmid pET28a-*yhgF*

18	GGGAAGCTTTTTCGTGCAAATTCGA	
19	CGCGGATCCATGAAAGGACAAGAAACTCGTGG	To construct plasmid pET28a-*htpG*
20	CCCAAGCTTTCAGGAAACCAGCAGCTGGTTC	
21	CGCGGATCCATGGGTAAAATAATTGGTATCG	To construct plasmid pET28a-*dnaK*
22	CGCGCCCAAGCTTTTATTTTTTGTCTTTGACTTCTTC	
23	TAATCTCCAGTAGCAACTTTGATCCGTTATGGGAGGAGTTGTGTAGGCTGGAGCTGCTTC	To construct the *clpB* deletion on chromosome
24	TTATAGACAAAAACGAGCCCCGAAGGGCTCGTTTTATCATCCATATGAATATCCTCCTTA	
25	CTATCCCCTGATTTTGATCCGAAAAGATGAACTCAAACCGTGTAGGCTGGAGCTGCTTC	To construct the *yhgF* deletion on chromosome
26	CGATTTCGTGCAAATTCGAACCGTAGGCCAGTACGGGCGTCCATATGAATATCCTCCTTA	
27	TGTTTTTAACCTTAAAATGGCATTATTGAGGTAGACCTACGTGTAGGCTGGAGCTGCTTC	To construct the *htpG* deletion on chromosome
28	AGAAAAATGCCGGATGACACGAAGGTCATCCGGCATTACACCATATGAATATCCTCCTTA	
29	ACAACCACATGATGACCGAATATATAGTGGAGACGTTTAGGTGTAGGCTGGAGCTGCTTC	To construct the *dnaK* deletion on chromosome
30	CCTTCGCCCGTGTCAGTATAATTACCCGTTTATAGGGCGACCATATGAATATCCTCCTTA	
31	GCGCGTGATTTTCTTTTCACATTAATCTGG	To check the *clpB* deletion on chromosome
32	CGGACTTTATCGTTCGCTCACTTTTCAG	
33	GAGCGTAAGGAAGAGTCTTAACCTCCTGC	To check the *yhgF* deletion on chromosome
34	ACTTTTATGTCAGGCAAGGCGTTCG	
35	AGCAGGATCACCTGCTCTCGCTTGA	To check the *htpG* deletion on chromosome
36	GGCACCTGGGGCTGAAACGGTTAAT	
37	GTCTGCAAAAAAATGAAATTGGG	To check the *dnaK* deletion on chromosome

	CA
38	TTGCTTAGCCATCTTTTTTAAATT<br>G
39	GAGCGCCTTCGCTTCGATCTGAC<br>GGATACGTTCGCGG

Supplementary Table S3 Primers used in RT-qPCR analysis.

ID No.	Sequence（5′---3′）	Gene
40	CTTACGACCAGGGCTACACAC	*16 S*
41	CGGACTACGACGCACTTTATG	
42	TTTAACCGACCGAACCGCAA	*ygaM*
43	CTGTACCCACGCTACACCAG	
44	CTATCCGCGGCTATGTCAGG	*fimF*
45	GCCGCTGACACCGTATTTC	
46	TAGCCTGACGAAAAACGCCT	*fimD*
47	TCCGCTATTTCCATCGCCTC	
48	TCTCTGGCAGCACAATCTGG	*eutA*
49	TTGATGACCGTCAGTACCGC	
50	CCCGGCGTTTAGTTATTGCG	*bglJ*
51	AAGCTGCTCGGCAATTTGTG	
52	TGTGGCCCGAAGAAAGTTCA	*yqeH*
53	ATTGCATATTGCGGTCGCTG	
54	CGACAGTGGTCGCCTTATCA	*ecpB*
55	GGGCCTGATCAAACCAGACA	
56	AGTTACTTTCGCAGTTCGGGT	*ariR*
57	GCTGTGTATCGCAACACGAT	
58	AGACAGCATTCTGACCTCGC	*leuO*
59	GAAACGATCGAGCGAAACCG	
60	AAACCAGGACCCGACCAATC	*flgD*
61	TTGCAGCTCAACACCAAACG	
62	AAAACTACCACCACGGCA	*flgE*
63	CCATCGCACCATCCACTA	
64	GCCACTGAACGCTTACACA	*flgG*
65	GACCACGCCATCACGAC	
66	AAAACTACCACCACGGCA	*fliE*
67	CCATCGCACCATCCACTA	
68	TTTTTGGTTGTGGATGACTT	*cheY*
69	GTTCTCTTTCTTCGCTTCTG	
70	TAACTGCCCAGTCCCAGC	*flgC*

71	CCATCTCTCCGACAACATCA	
72	GCGTGAGCAGTGAAAGCC	*rhsD*
73	CCCATCGGGTCCTGAGTAA	
74	CGATGGGGTTGAAAGGG	*flu*
75	GGTGTTGCGAGATAAAATGC	
76	GGAGCATCATAGCAATAAA	*ybgD*
77	CGAAAAAGTTCAGCGTC	
78	TCCGCACTCATCACTGTCG	*srlM*
79	GGCTGTAAATCACCCGCAT	
80	ATCAGTGGTCTGGTTGTTG	*yadN*
81	GAAGGTCATTTTAGCCGT	
82	AACCACACCAAGTCTATCGG	*purR*
83	GCTTTTGCTTCACCCCA	
84	TACCCAGTTTCACATCTCCC	*allS*
85	GCTTCTGTTAGCGGCTCTT	
86	CCACGATGCTTCTAACA	*rcsA*
87	CACATTCGCAACATACTC	
88	GGTCGGTTCTGTAGAGGTG	*torZ*
89	GGTTTCGGAGCGGATAG	
90	TGGCGGAAGGGAAAAT	*mfd*
91	GGACCACCATGCTGTCATA	
92	TCTTGATGGCGTGGACT	*hchA*
93	ACAACGATTTATGCTGCTC	
94	TGGAATGCCAACCTCG	*chiP*
95	GAATGTAACCTGCCCTCG	
96	TTATTCACCTCTCGCAGCCT	*essD*
97	ATGAAATCCATGGATAAGTT	
98	GTGGCTTCCTGTTCAAAAT	*ptsG*
99	TACCGTCACGCATCCC	
100	CCTGATTTTGGTTGTCTTC	*psiE*
101	ACACATCCAGTGGCGAT	
102	TCATCATCGGTGGTCTGG	*yidE*
103	GGAAAATAACCCGCAACAT	
104	GGCGGGGCTTTGTGGGGTTA	*menH*
105	TCAGGCTGGACGGCGAGAGA	
106	CATTATTTTGCTTTGCCAGT	*ydbL*
107	CATTATTTTGCTTTGCCAGT	
108	GGGTTCGGTTAGTCCTTT	*clpB*

109	CGCTTGAGTAATGTTGGC	
110	CCGATCCACAGTTCGATGA	*yhgF*
111	TTACCCCAGTACGCAGACC	
112	GGCGAAGACGAGTTCCT	*htpG*
113	TGGGCGATGTGTTTGTAG	
114	CCTCGTTGGTGGTCAGA	*dnaK*
115	GTGCTTGGTCGGGATAG	

Supplementary Table S4 The proteins pulled-down with RpoD in RpoD-GFP-Trap immunoprecipitation.

Protein	Function	Score
FusA	Elongation factor G	2456
NarG	Respiratory nitrate reductase 1 alpha chain	2152
PTA	Phosphate acetyltransferase	1979
AcnB	Aconitate hydratase 2	1740
ClpB	Chaperone protein ClpB	1442
InfB	Translation initiation factor IF-2	1356
YhgF	Protein YhgF	970
YnfF	Probable dimethyl sulfoxide reductase chain YnfF	881
DnaK	Chaperone protein DnaK	873
NrdA	Ribonucleoside-diphosphate reductase 1 subunit alpha	849
AdhE	Aldehyde-alcohol dehydrogenase	805
GyrA	DNA gyrase subunit A	798
AlaS	Alanine--tRNA ligase	796
AceF	Dihydrolipoyllysine-residue acetyltransferase component of pyruvate dehydrogenase complex	792
TufA	Elongation factor Tu	781
PutA	Bifunctional protein PutA	769
SucA	2-oxoglutarate dehydrogenase E1 component	744
CAT	Chloramphenicol acetyltransferase	729
LeuS	Leucine--tRNA ligase	708
IleS	Isoleucine--tRNA ligase	693
Lon	Lon protease	673
GroL	60 kDa chaperonin	659
NuoH	NADH-quinone oxidoreductase subunit H	583
PepN	Aminopeptidase N	546
BamA	Outer membrane protein assembly factor BamA	524
PflB	Formate acetyltransferase 1	519
ValS	Valine--tRNA ligase	493

MukB	Chromosome partition protein MukB	483
Lpp	Major outer membrane lipoprotein Lpp	403
RpsA	30S ribosomal protein S1	386
RapA	RNA polymerase-associated protein RapA	359
GatC	Galactitol permease IIC component	344
LepA	Elongation factor 4	310
GyrB	DNA gyrase subunit B	310
RpsC	30S ribosomal protein S3	263
RplB	50S ribosomal protein L2	234
AdhP	Alcohol dehydrogenase, propanol-preferring	218
TnaA	Tryptophanase	215
MreB	Rod shape-determining protein MreB	203
ThrA	Bifunctional aspartokinase/homoserine dehydrogenase 1	192
RplE	50S ribosomal protein L5	189
PntA	NAD（P） transhydrogenase subunit alpha	183
Pnp	Polyribonucleotide nucleotidyltransferase	181
RplO	50S ribosomal protein L15	178
Mdh	Malate dehydrogenase	175
Ppc	Phosphoenolpyruvate carboxylase	172
CarB	Carbamoyl-phosphate synthase large chain	172
GcvP	Glycine dehydrogenase [decarboxylating]	169
RecJ	Single-stranded-DNA-specific exonuclease RecJ	158
HtpG	Chaperone protein HtpG	153
PpsA	Phosphoenolpyruvate synthase	152
RpoS	RNA polymerase sigma factor RpoS	150
TopA	DNA topoisomerase 1	143
Prc	Tail-specific protease	140
RplD	50S ribosomal protein L4	133
CysK	Cysteine synthase A	129
MutS	DNA mismatch repair protein MutS	115
PtsI	Phosphoenolpyruvate-protein phosphotransferase	110
SerS	Serine--tRNA ligase	109
OmpC	Outer membrane protein C	104
GlpK	Glycerol kinase	101
GapA	Glyceraldehyde-3-phosphate dehydrogenase A	100

Supplementary Table S5 The 4 DEGs associated with DNA replication in Δ*dnaK* mutant.

Gene	log^2 Fold Change	Fuction

	Up-regulation		
	LB	ABTGcasa	
dnaQ	1.0167		DNA polymerase III epsilon subunit
dinB	1.7787	1.6453	DNA polymerase IV
polB		1.1994	DNA polymerase II
umuD		1.7319	translesion error-prone DNA polymerase V subunit RecA-activated auto-protease

Supplementary Table S6 The components of 1 L ABTGcasa and LB medium.

Component	Volume		
ABTGcasa			
		Component	Mass（g）
5×A	200 mL	（NH4）$_2SO_4$	10
		Na_2HPO_4	30
		KH_2PO_4	15
		NaCl	15
		ddH_2O	Add to 1 L
0.003 M $FeCl_3$	1 mL		
1 M $MgCl_2$	1 mL		
0.1 M $CaCl_2$	1 mL		
10 mg/mL V_{B1}	1 mL		
20% Glucose	10 mL		
10% Casamino acid	50 mL		
ddH_2O	736 mL		
LB			
	Mass（g）		
Tryptone	10 g		
Yeast extract	5 g		
NaCl	10 g		
ddH2O	Up to 1 L		

Supplementary Table S7 Sequencing data statistics.

Sample name	Raw reads	Clean reads	Error rate	Q20	Q30	GC content	Uniq Mapped Reads Ratio (%)
LB_Wt_1	3532000	3505329	0.01%	99.12%	97.07%	51.98%	94.24%
LB_Wt_2	3532000	3505329	0.01%	97.56%	93.95%	51.67%	
LB_delB_1	3547560	3523185	0.01%	99.12%	97.04%	52.41%	94.29%
LB_delB_2	3547560	3523185	0.01%	97.59%	93.98%	52.10%	
LB_delF_1	3775250	3753050	0.01%	99.24%	97.40%	52.31%	92.05%
LB_delF_2	3775250	3753050	0.01%	97.89%	94.67%	52.07%	
LB_delG_1	4638723	4608374	0.01%	99.21%	97.25%	52.29%	95.1%
LB_delG_2	4638723	4608374	0.01%	97.70%	94.21%	51.95%	
LB_delK_1	4654014	4626493	0.01%	99.19%	97.25%	51.74%	92.96%
LB_delK_2	4654014	4626493	0.01%	97.78%	94.44%	51.46%	
Ac_Wt_1	3047629	3041887	0.02%	98.56%	95.75%	52.21%	93.21%
Ac_Wt_2	3047629	3041887	0.02%	97.99%	94.53%	52.08%	
Ac_delB_1	4645927	4626472	0.01%	99.13%	97.14%	52.32%	94.16%

Ac_delB_2	4645927	4626472	0.01%	98.18%	95.37%	51.98%	
Ac_delF_1	3313706	3292317	0.01%	99.14%	97.07%	52.34%	94.56%
Ac_delF_2	3313706	3292317	0.01%	97.56%	93.87%	52.03%	
Ac_delG_1	3459254	3436736	0.01%	99.13%	97.06%	52.44%	95.89%
Ac_delG_2	3459254	3436736	0.01%	97.55%	93.87%	52.10%	
Ac_delK_1	4626472	4599051	0.01%	99.17%	97.17%	52.32%	93.17%
Ac_delK_2	4626472	4599051	0.01%	97.74%	94.29%	51.98%	

(1) Raw reads: the number of reads of the original sequencing data; (2) Clean reads: the number of reads obtained after the quality control is completed; (3) Error rate: the average sequencing error rate of clean reads; (4) Q20 and Q30: the proportion of base numbers with phred mass values greater than 20 and 30 to the total base number (clean data); (5) GC content: the proportion of GC in the sequencing data to the total base number (clean data); (6) Uniq Mapped Reads Ratio (%) : percentage of clean reads that are aligned to a unique position in the reference genome (GCF_000750555.1).
