# Supplementary material for: The Roles of Molecular Chaperones Interacting with the σ70 Factor in Global Transcription of the Escherichia coli Genome
Source: Genes (Basel). 2026 May 29;17(6):621. doi: 10.3390/genes17060621 (PMC13298495; doi:10.3390/genes17060621)
Supplement: Supplementary file 1 [file genes-17-00621-s001.zip › Supplementary Figures.pdf]

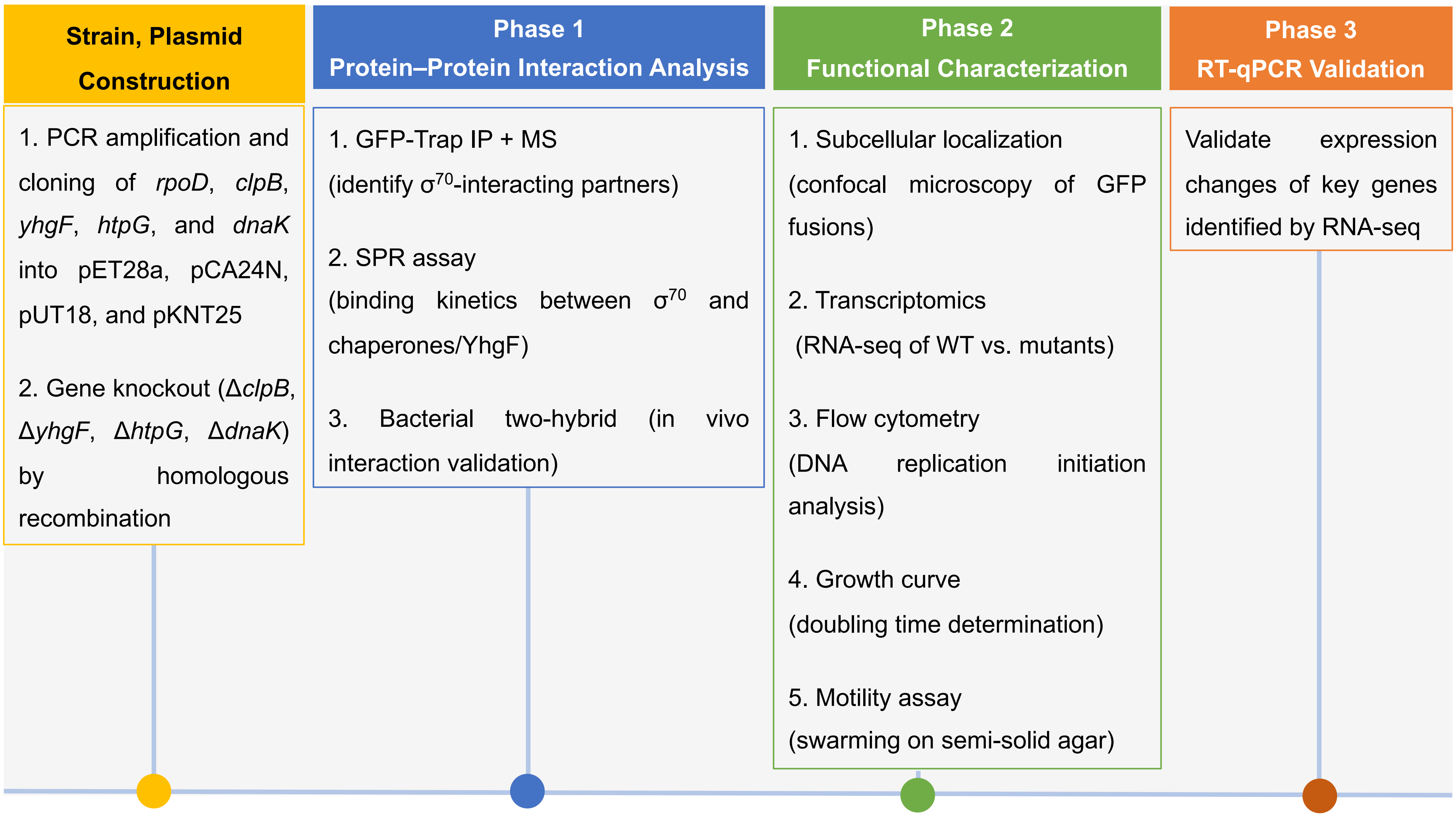

Study workflow timeline: A schematic representation of the sequential experimental procedures performed in this study. Strain construction and plasmid preparation. Phase 1: Protein–protein interaction analyses (GFP–Trap IP/MS, SPR, bacterial two-hybrid). Phase 2: Functional characterizations (subcellular localization, transcriptomics, flow cytometry, growth curve, motility assay). Phase 3: RT-qPCR validation of RNA-seq findings.

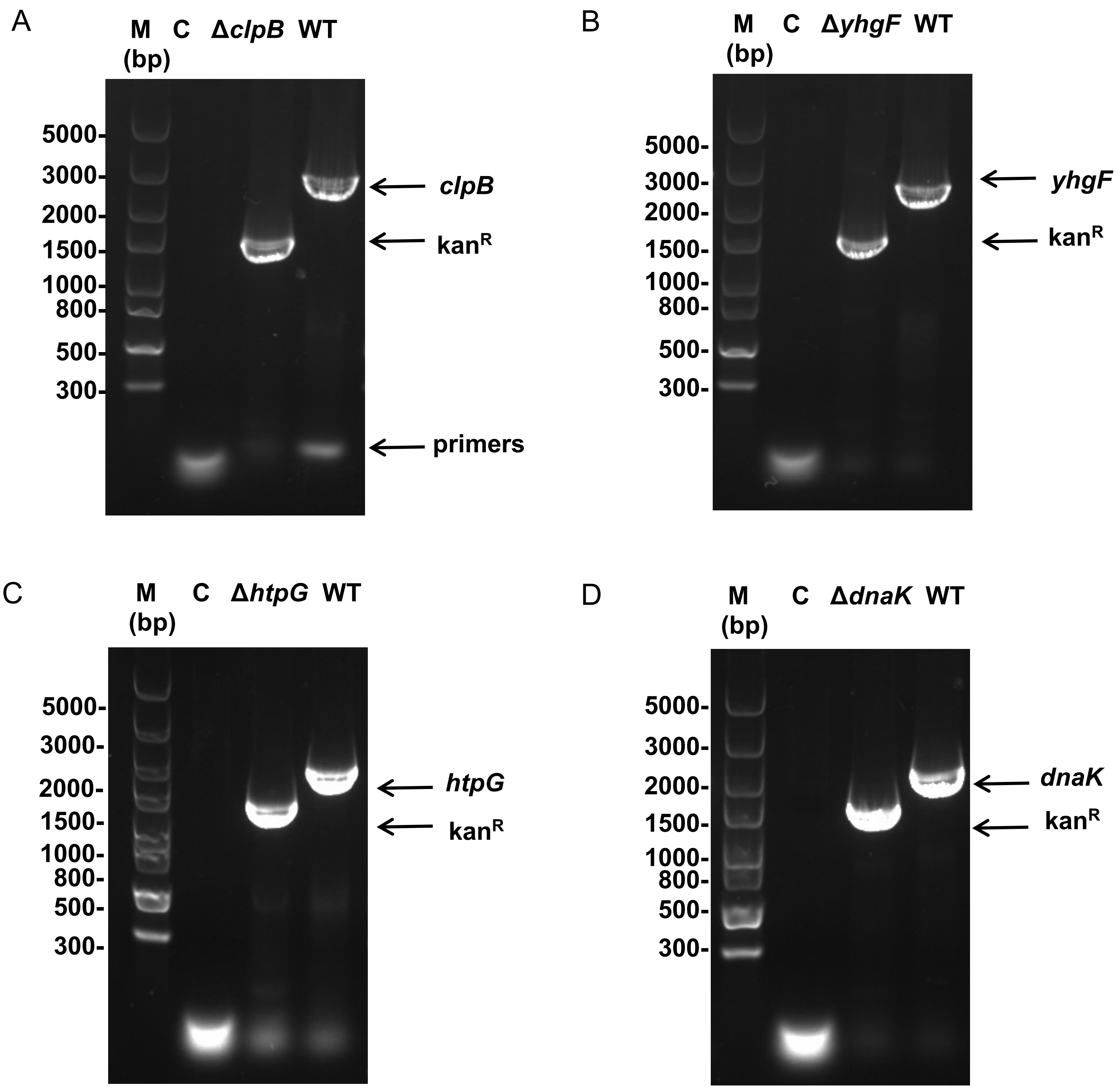

Supplement Figure S1: Characterizations of Δ*clpB*, Δ*yhgF*, Δ*htpG*, and Δ*dnaK* mutants by PCR technique. To confirm the correctness in construction of BW25113Δ*clpB*::*neo* (*kan*^{R}) (A), BW25113Δ*yhgF*::*neo* (*kan*^{R}) (B), BW25113Δ*htpG*::*neo* (*kan*^{R}) (C), or BW25113Δ*dnaK*::*neo* (*kan*^{R}) (D) mutant, PCR amplification was performed using genomic DNA from each mutant as a template. Primers target regions upstream and downstream of the respective genes (primer 23-30 listed in Supplementary Table 2) were used, and the resulting PCR fragments were analyzed by agarose gel electrophoresis. When the construction is correct, then the *neo* (*kan*^{R}) gene fragment (100-200 bp bigger than the coding region of *kan*^{R}) will be amplified due to a replacement of the target gene by *kan*^{R}. Otherwise, the target gene will be amplified as found in wild type (WT) cells. The experiment includes a negative control where water was used as a template.

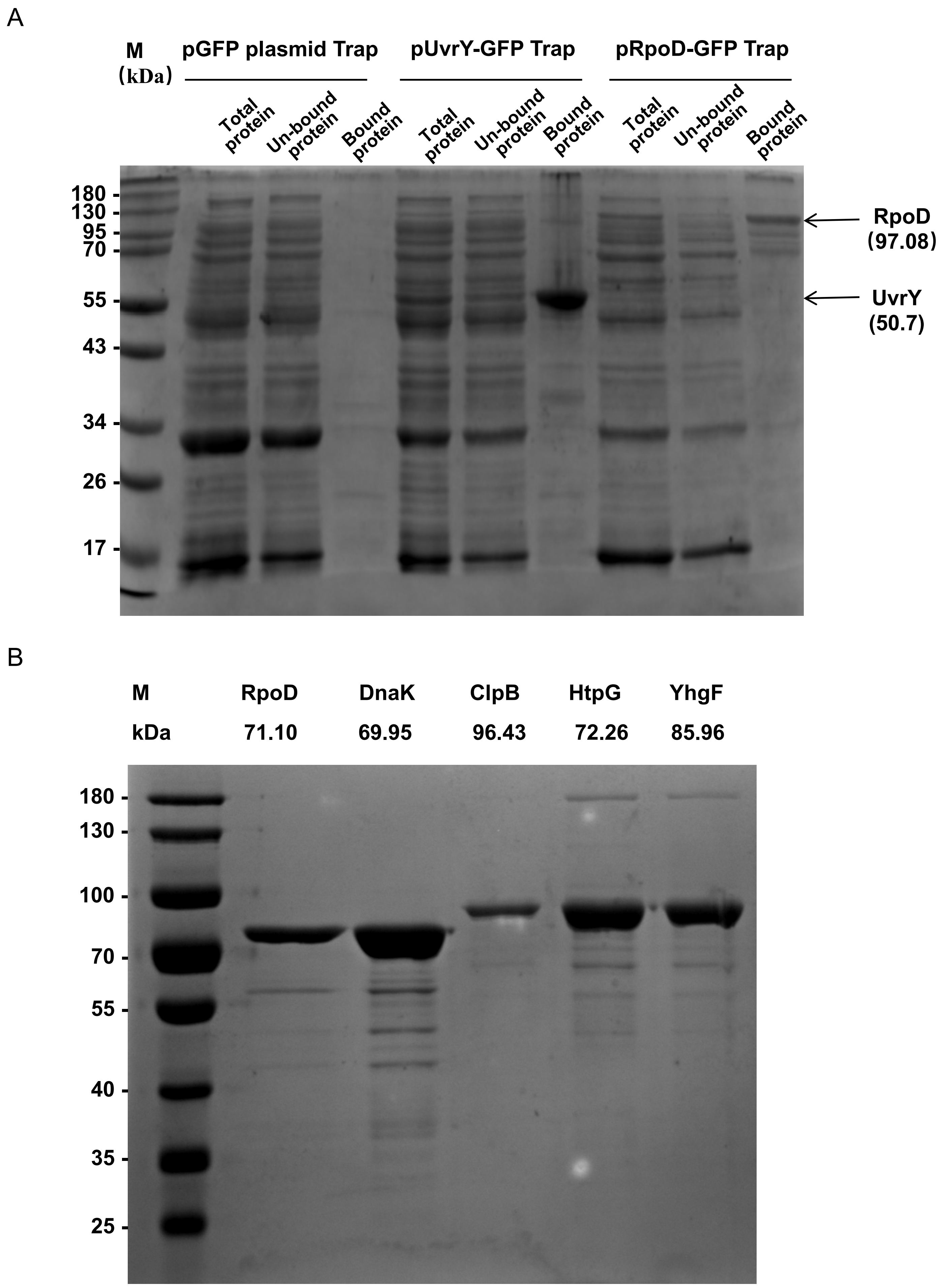

Supplement Figure S2: Co-immunoprecipitation with RpoD and protein purification. (A) Cells carrying pCA24N-RpoD-GFP, pCA24N-GFP (negative control), or pCA24N-UvrY-GFP (positive control) were cultured exponentially in LB with required antibiotics. The cells were harvested and lysed. The lysates were immunoprecipitated by using GFP-Trap technique, as described in Materials and Methods, and then subjected to SDS-PAGE. Each of the three lysates include total protein, unbound protein and bound protein lanes as indicated. The gel also includes a lane of protein marker. The RpoD-GFP and UvrY-GFP are as shown on right side of the gel. The pCA24N-UvrY-GFP plasmid generates a fusion protein of 456 amino acids (238 from GFP and 218 from UvrY), and molecular weight of 50.7 kDa. The pCA24N-RpoD-GFP plasmid produces a fusion protein of 856 amino acids (238 from GFP and 618 from RpoD), with molecular weight of 97.08 kDa. (B) RpoD, ClpB, YhgF, HtpG, and DnaK were purified. BL21(DE3) cells carrying pET28a-*rpoD*, pET28a-*clpB*, pET28a-*yhgF*, pET28a-*htpG*, or pET28a-*dnaK* were exponentially grown in LB to express the recombinant protein with His-tag. The recombinant proteins with His-tag were purified by nickel affinity chromatography (Ni-NTA). The purified proteins were subjected to SDS-PAGE, names of proteins are as indicated on top of the gel. The final recombinant His 6 -tagged RpoD, ClpB, HtpG, YhgF, and DnaK protein consists of 619, 863, 630, 779, and 644 amino acid residues, with molecular weight of approximately 71.10 kDa, 96.43 kDa, 72.26 kDa, 85.96 kDa, and 69.95 kDa.

pCA24N/BW25113

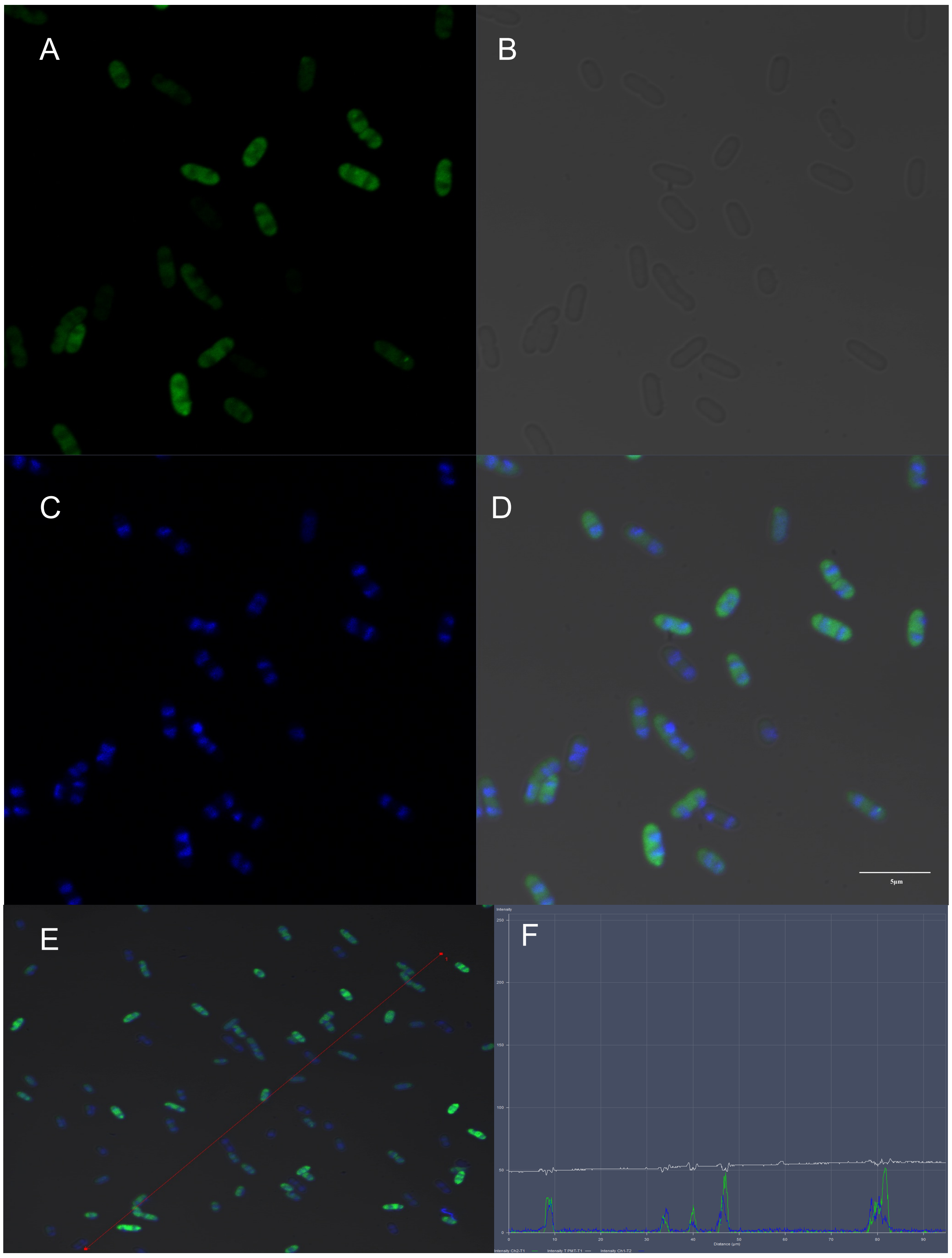

Supplement Figure S3: No co-localization of GFP empty vector (pCA24N) with the nucleoids in BW25113. The cells were exponentially cultured at 37℃ in ABTGcasa medium and a final concentration of 0.1 mM IPTG was added to the culture when OD_{450} reached to 0.1. The cell nucleoids were stained with Hoechst33258 (blue) in all experiments. The green fluorescence from GFP (A), brightfield cells (B), blue from Hoechst33258 (C), overlap of green and blue fluorescence (D, E), fluorescence intensity histogram (F) was visualized and analyzed using confocal fluorescence microscope (ZEISS, LSM710, Germany) (100× objective) and ZEISS software, respectively.

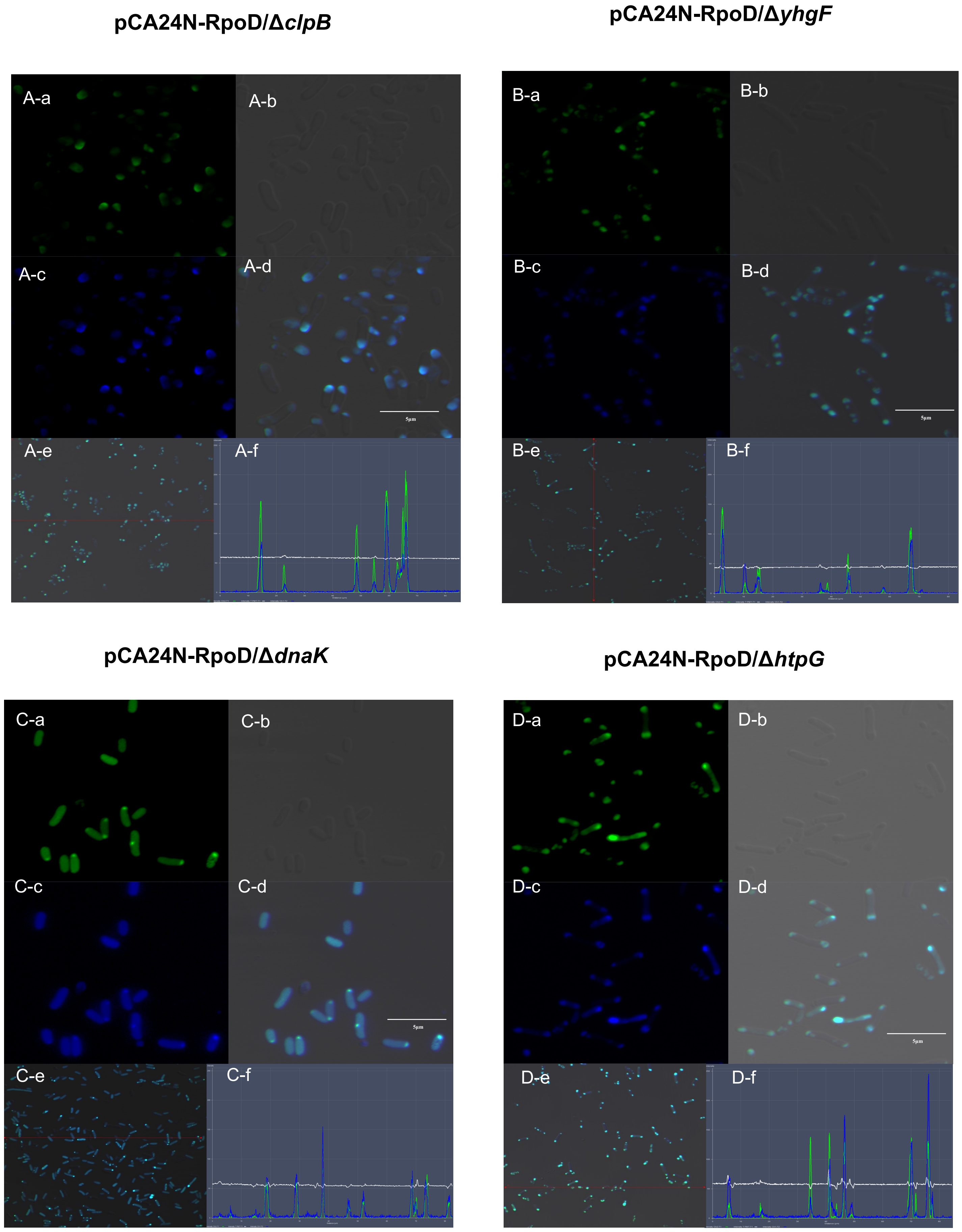

Supplement Figure S4: RpoD, ClpB, YhgF, HtpG, and DnaK co-localize with nucleoids. pCA24N-*rpoD*-gfp/BW25113 (A), pCA24N-*clpB*-gfp/Δ*clpB* (B), pCA24N-*yhgF*-gfp/Δ*yhgF* (C), pCA24N-*htpG*-gfp/Δ*htpG* (D), or pCA24N-*dnaK*-gfp/Δ*dnaK* (E) cells were exponentially grown at 37℃ in LB medium with induction of IPTG (0.1 mM). The cells were harvested and fixed in 70% ethanol after a wash, and nucleoids were stained with Hoechst33258 (blue). The green fluorescence from GFP (-a), brightfield cells (-b), blue from Hoechst33258 (-c), overlap of green and blue fluorescence (-d, -e), fluorescence intensity histogram (-f) were visualized and analyzed using confocal fluorescence microscope (ZEISS, LSM710, Germany) (100× objective) and ZEISS software. (e) A wider field of view at lower magnification. The red line indicates the position used for the line-scan analysis of fluorescence intensity shown in panel (f).

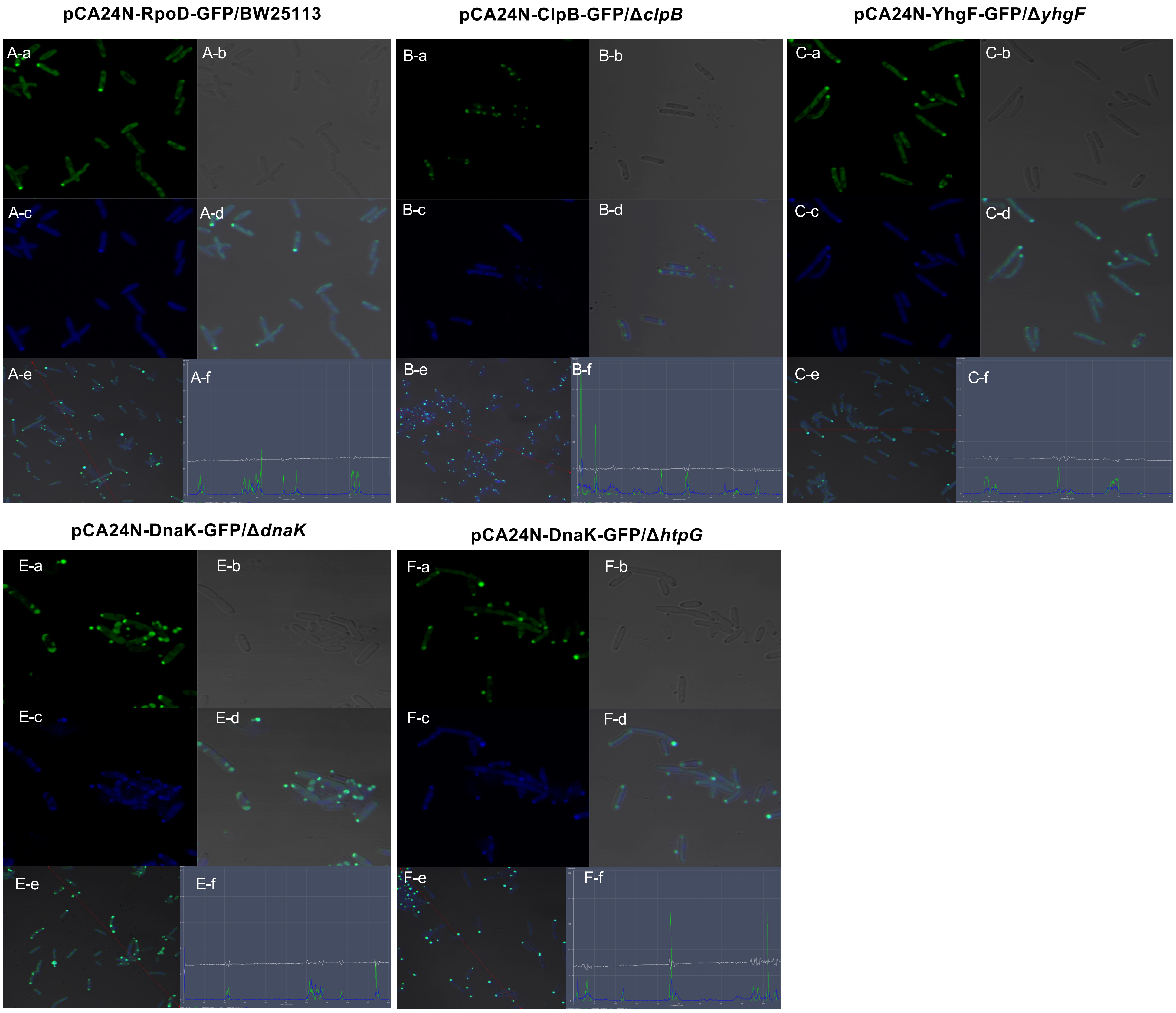

Supplement Figure S5: Co-localization of RpoD with the nucleoids in ΔclpB (A), ΔyhgF (B), ΔhtpG (C), and ΔdnaK (D) mutants in ABTGcasa. The green fluorescence from GFP (-a), brightfield cells (-b), blue from Hoechst33258 (-c), overlap of green and blue fluorescence (-d, -e), fluorescence intensity histogram (-f) were visualized and analyzed using confocal fluorescence microscope (ZEISS, LSM710, Germany) (100× objective) and ZEISS software.

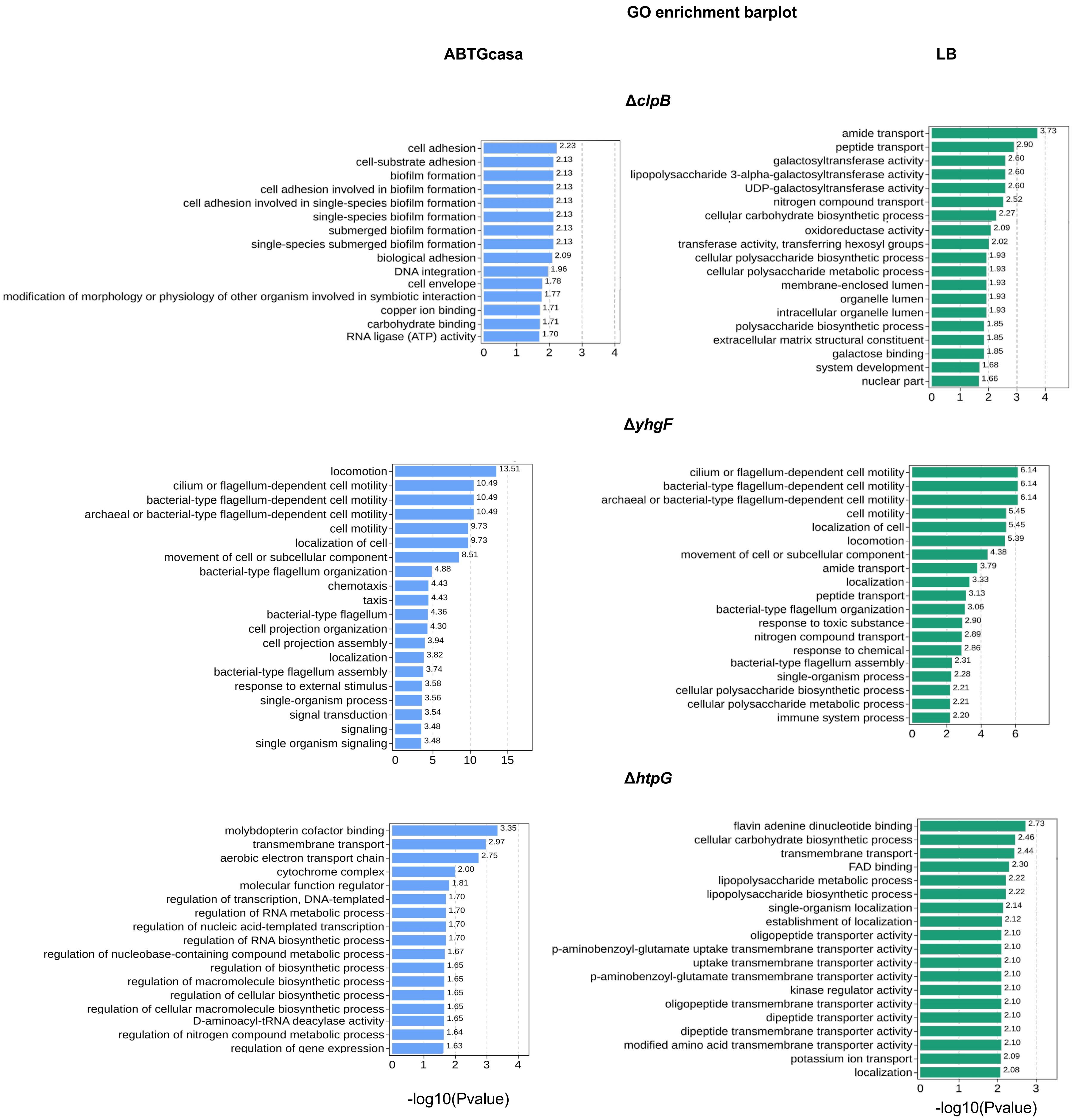

Supplement Figure S6: GO enrichment analysis of DEGs in Δ*clpB*, Δ*yhgF*, and Δ*htpG* mutants. The growth conditions and data collection are as mentioned in the legend to Figure 3. The DEGs were identified by using criteria of P value < 0.05, |log FC| > 1. The cellular processes in which the DEGs are involved in, and genotype of the cells are as indicated.

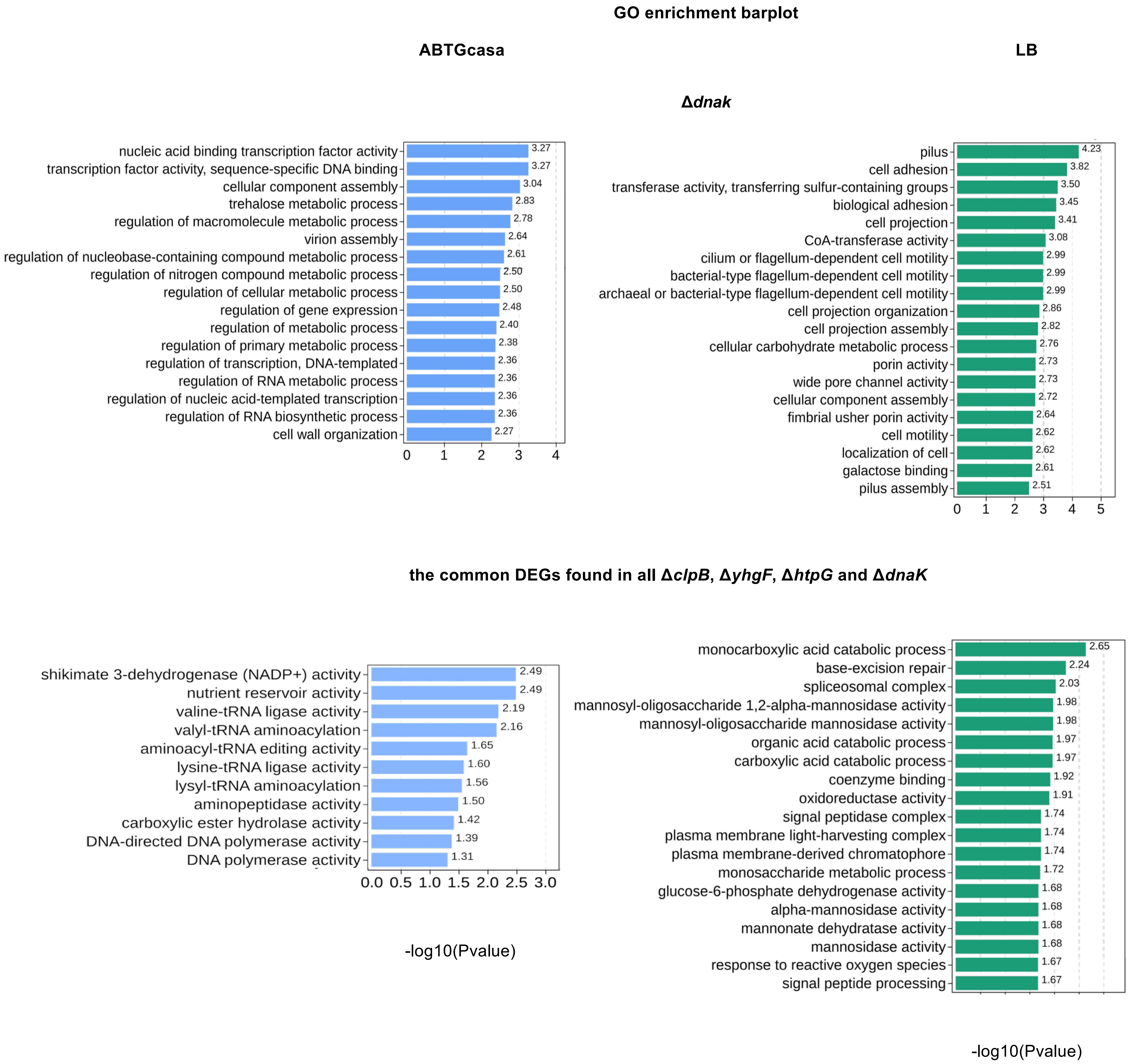

Supplement Figure S7: GO pathway enrichment analyses of DEGs in Δ*dnaK* mutant and the common DEGs found in Δ*clpB*, Δ*yhgF*, Δ*htpG*, and Δ*dnaK* mutants. The DEGs were identified by using criteria of P value < 0.05, |log FC| > 1. The cellular processes in which the DEGs are involved in and up- or down-regulation of DEGs in each growth medium are as indicated.

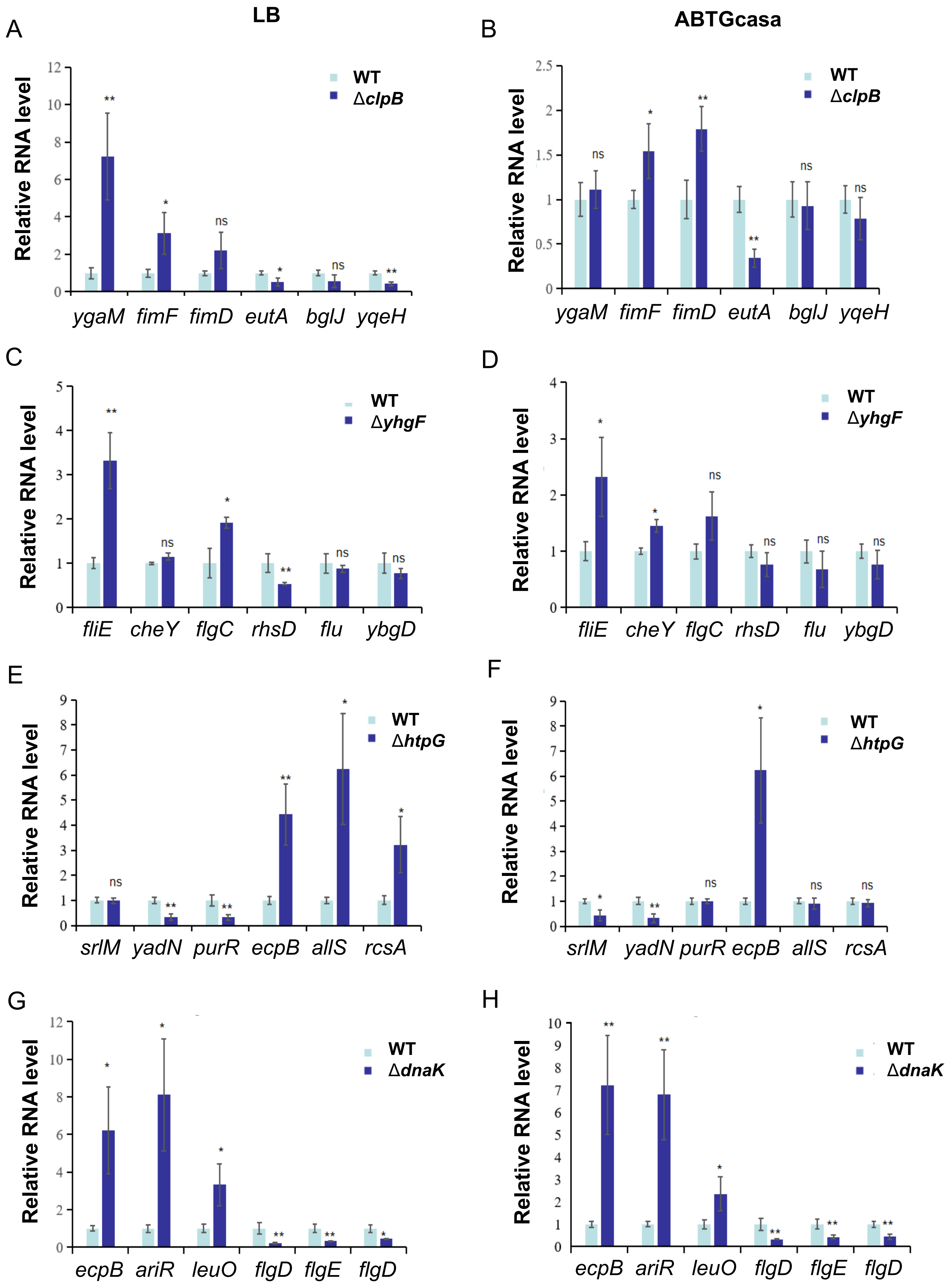

Supplement Figure S8: Relative RNA levels of the chosen DEGs in Δ*clpB*, Δ*yhgF*, Δ*htpG*, or Δ*dnaK* mutants. To confirm the RNA-seq results, the expression levels of selected DEGs were measured by RT-qPCR in each mutant strain grown exponentially in either LB or ABTGcasa medium, relative to WT. Different sets of DEGs were analyzed for Δ*clpB* (A, B), Δ*yhgF* (C, D), Δ*htpG* (E, F), or Δ*dnaK* (G, H). Data are presented as mean ± SD from three independent experiments. Statistical significance was determined using a two-tailed paired t-test ($^{*}p$ <0.05, $^{**}p$ <0.01, and ^{ns}p>0.05).

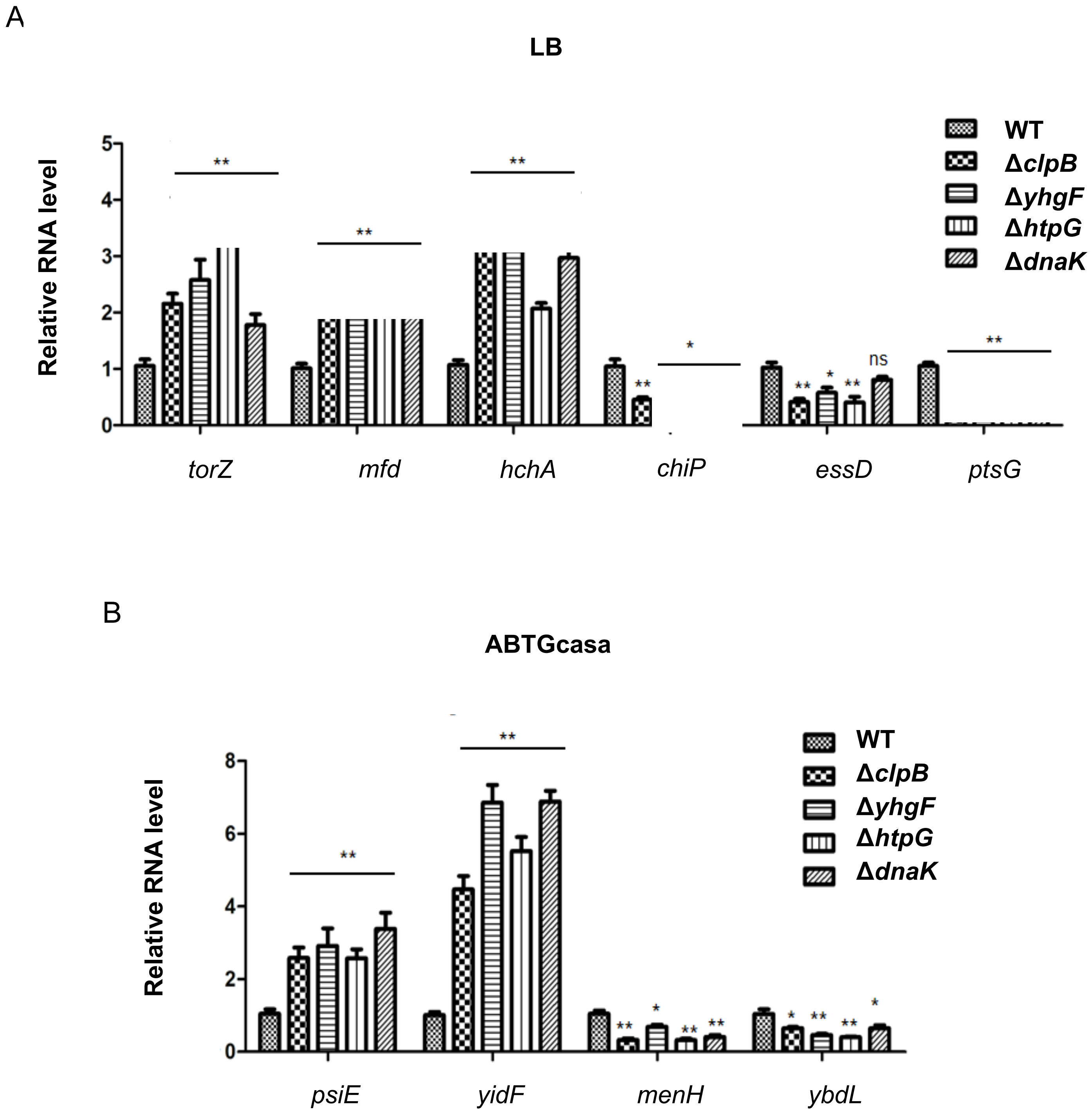

Supplement Figure S9: Relative RNA levels of the common DEGs in Δ*clpB*, Δ*yhgF*, Δ*htpG*, and Δ*dnaK* mutants. (A) RT-qPCR analysis was performed to determine the RNA levels of six common DEGs in cells grown exponentially in LB medium (A) and four common DEGs in cells grown in ABTGcasa medium (B), relative to WT. Values represent the mean ± SD from three independent experiments. Statistical significance was assessed using a two-tailed, paired Student's t-test ($^{*}p < 0.05$; $^{**}p < 0.01$).

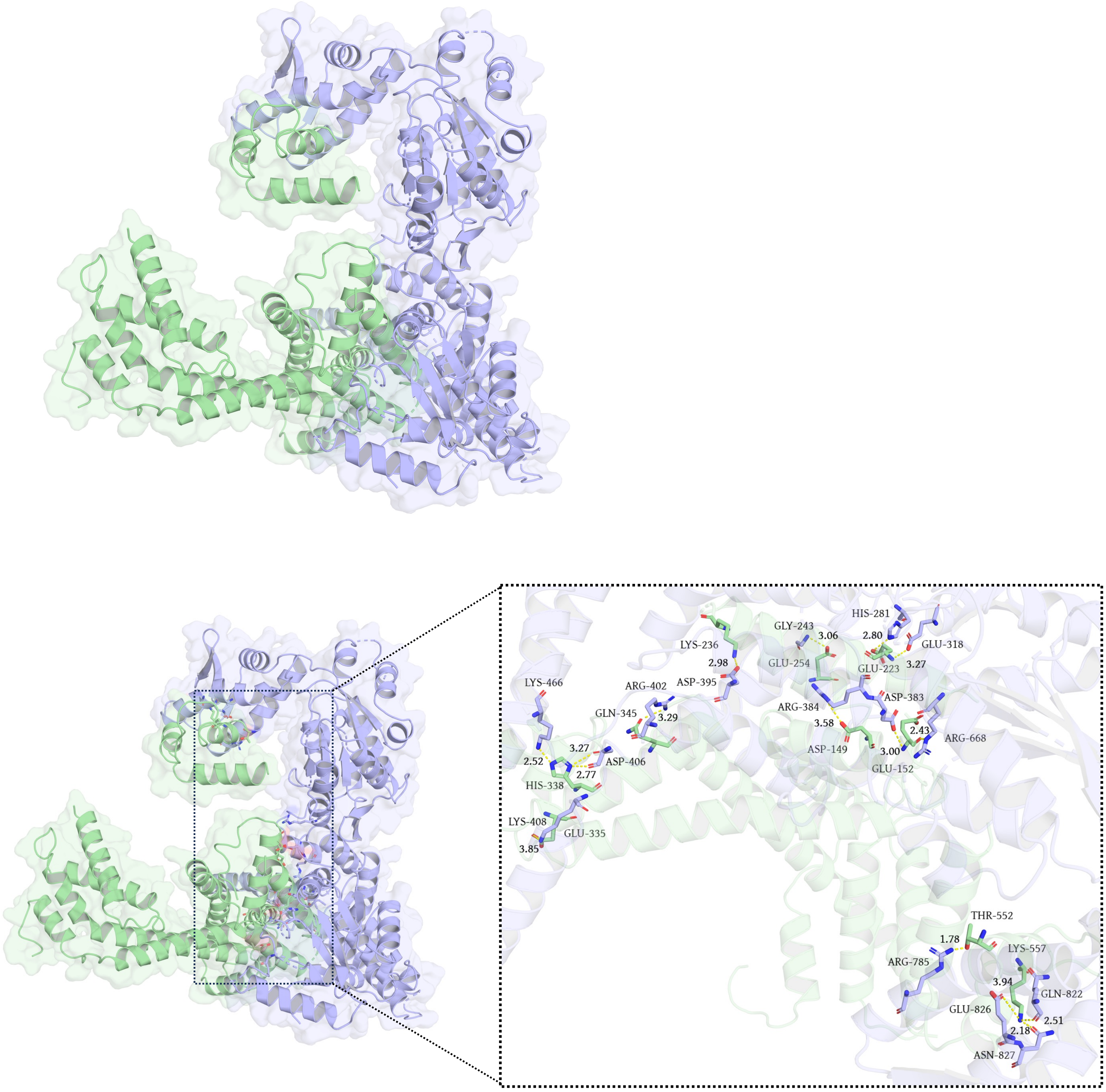

Supplement Figure S10: Molecular interactions between RpoD and ClpB. RpoD is shown in green and ClpB in purple. Key interacting residues and hydrogen bond distances (Å) are indicated.

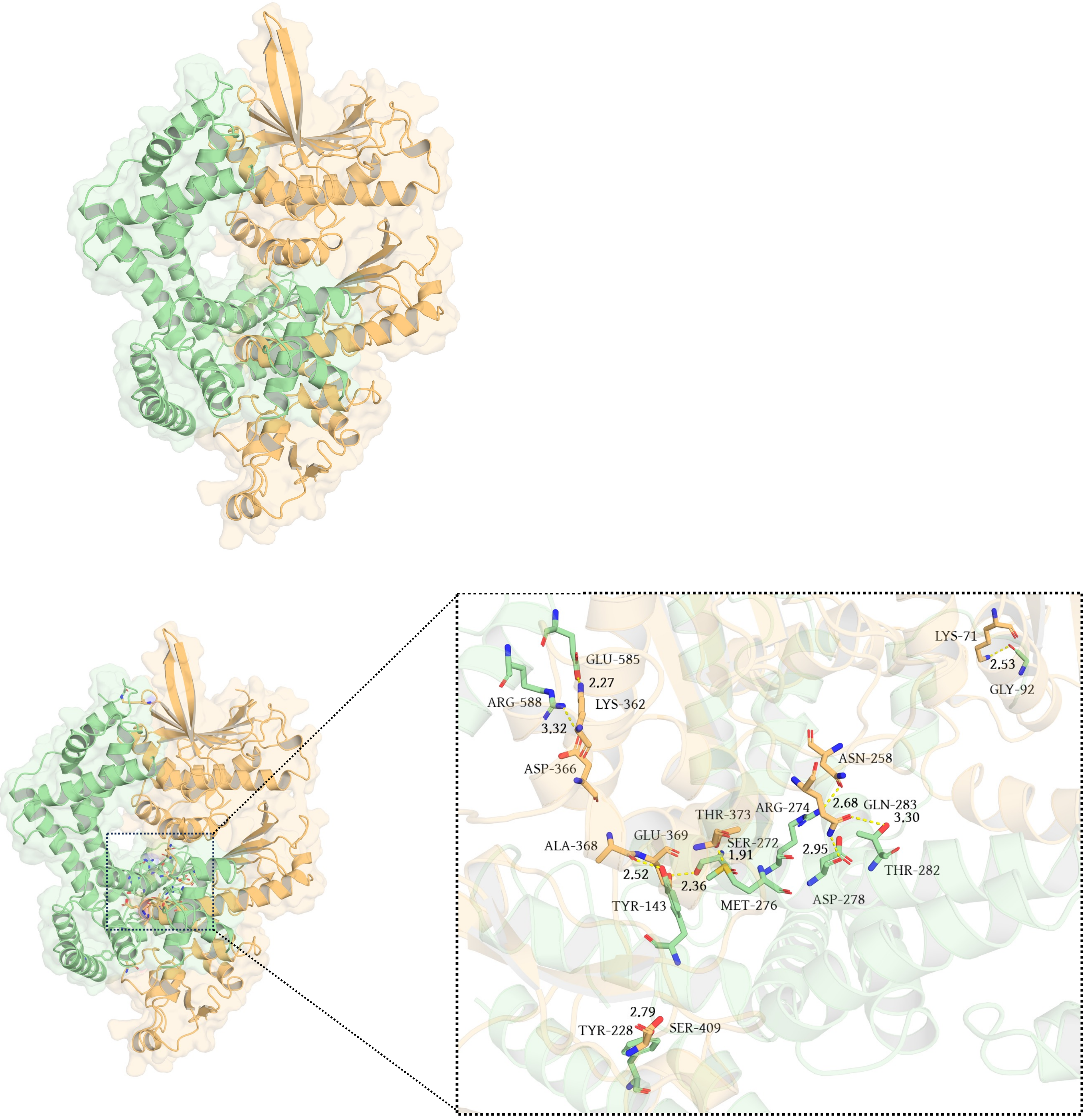

Supplement Figure S11: Molecular interactions between RpoD and HtpG. RpoD is shown in green and HtpG in Orange. Key interacting residues and hydrogen bond distances (Å) are indicated.

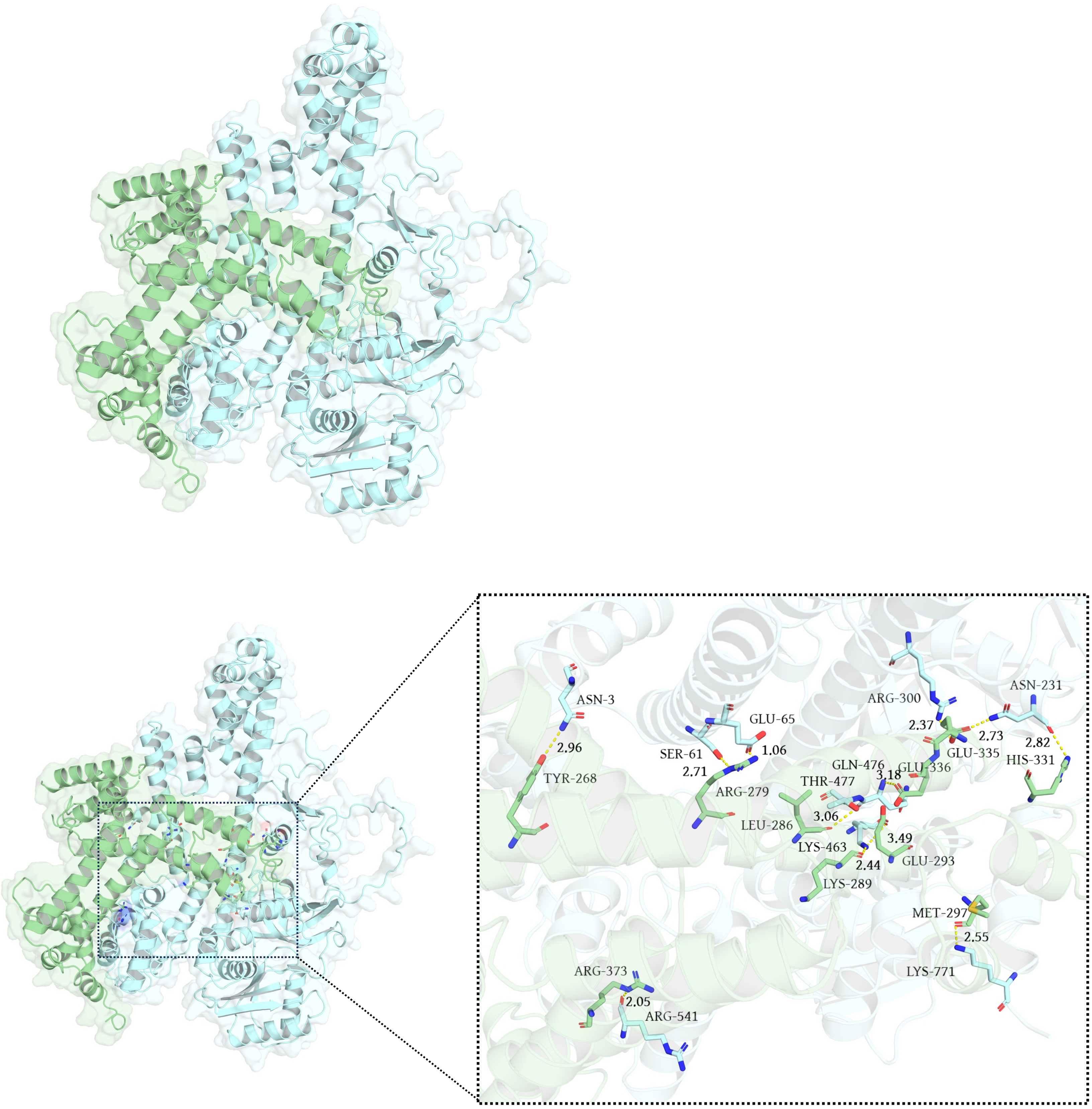

Supplement Figure S12: Molecular interactions between RpoD and YhgF. RpoD is shown in green and YhgF in blue. Key interacting residues and hydrogen bond distances (Å) are indicated.

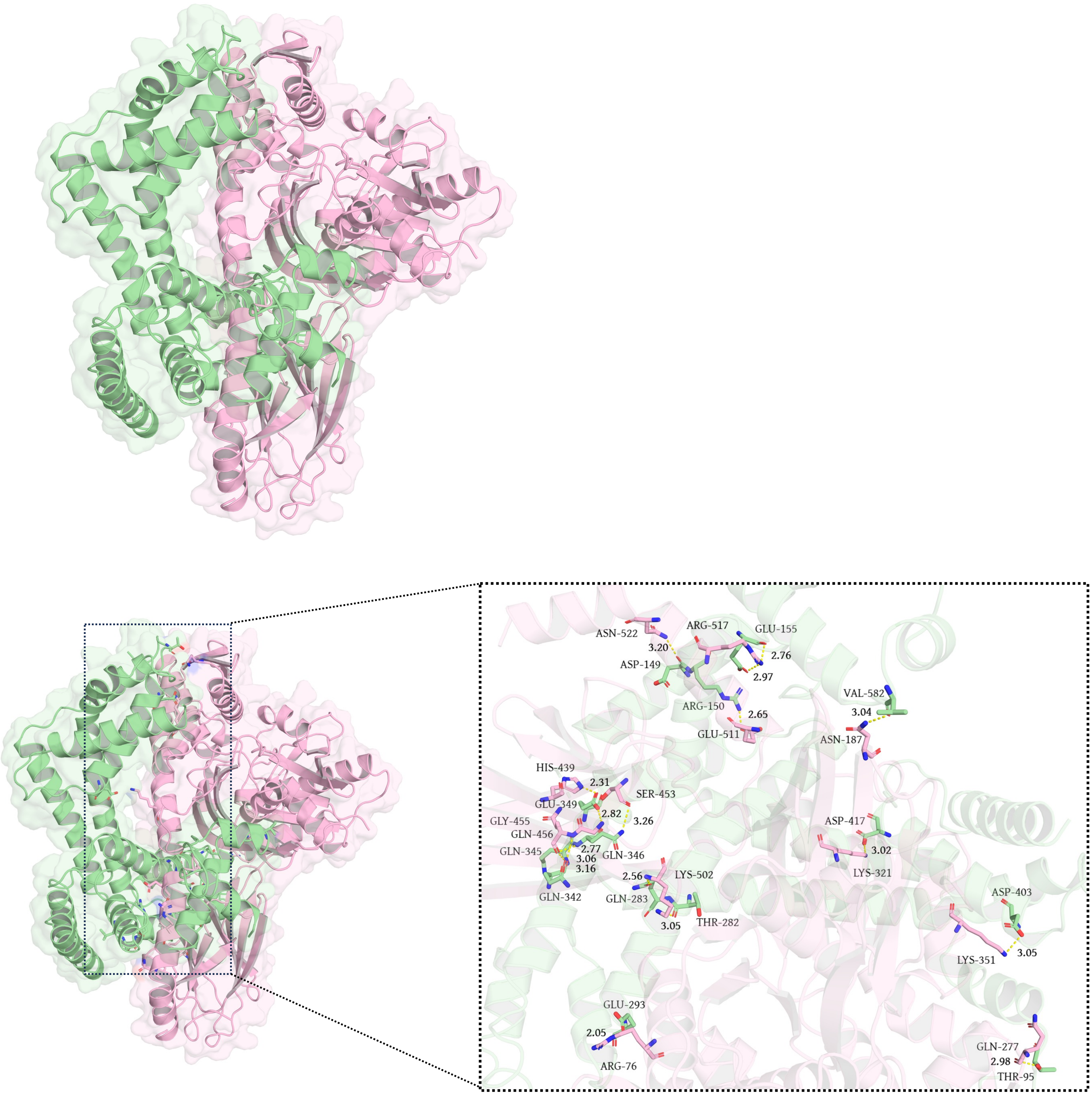

Supplement Figure S13: Molecular interactions between RpoD and DnaK. RpoD is shown in green and DnaK in pink. Key interacting residues and hydrogen bond distances (Å) are indicated.
